# BiœmuS: A new tool for neurological disorders studies through real-time emulation and hybridization using biomimetic Spiking Neural Network

**DOI:** 10.1038/s41467-024-48905-x

**Published:** 2024-06-20

**Authors:** Romain Beaubois, Jérémy Cheslet, Tomoya Duenki, Giuseppe De Venuto, Marta Carè, Farad Khoyratee, Michela Chiappalone, Pascal Branchereau, Yoshiho Ikeuchi, Timothée Levi

**Affiliations:** 1 IMS, CNRS UMR5218, Bordeaux INP, University of Bordeaux, Talence, France; 2 Institute of Industrial Science, The University of Tokyo, Tokyo, Japan; 3 LIMMS, CNRS-Institute of Industrial Science, UMI 2820, The University of Tokyo, Tokyo, Japan; 4 Department of Chemistry and Biotechnology, Graduate School of Engineering, The University of Tokyo, Tokyo, Japan; 5 Institute for AI and Beyond, The University of Tokyo, Tokyo, Japan; 6 DIBRIS, University of Genova, Genova, Italy; 7 IRCCS Ospedale Policlinico San Martino, Genova, Italy; 8 Rehab Technologies, Istituto Italiano di Tecnologia, Genova, Italy; 9 INCIA, UMR5287, CNRS, University of Bordeaux, Bordeaux, France

**Keywords:** Biomedical engineering, Biophysical models, Dynamical systems, Electrical and electronic engineering

## Abstract

Characterization and modeling of biological neural networks has emerged as a field driving significant advancements in our understanding of brain function and related pathologies. As of today, pharmacological treatments for neurological disorders remain limited, pushing the exploration of promising alternative approaches such as electroceutics. Recent research in bioelectronics and neuromorphic engineering have fostered the development of the new generation of neuroprostheses for brain repair. However, achieving their full potential necessitates a deeper understanding of biohybrid interaction. In this study, we present a novel real-time, biomimetic, cost-effective and user-friendly neural network capable of real-time emulation for biohybrid experiments. Our system facilitates the investigation and replication of biophysically detailed neural network dynamics while prioritizing cost-efficiency, flexibility and ease of use. We showcase the feasibility of conducting biohybrid experiments using standard biophysical interfaces and a variety of biological cells as well as real-time emulation of diverse network configurations. We envision our system as a crucial step towards the development of neuromorphic-based neuroprostheses for bioelectrical therapeutics, enabling seamless communication with biological networks on a comparable timescale. Its embedded real-time functionality enhances practicality and accessibility, amplifying its potential for real-world applications in biohybrid experiments.

## Abstract (web summary)

Beaubois et al. introduce a real-time biomimetic neural network for biohybrid experiments, providing a tool to study closed-loop applications for neuroscience and neuromorphic-based neuroprostheses.

## Introduction

Millions of people worldwide are affected by neurological disorders that strongly impair their cognitive and/or motor functions^1^. An increasing number of technologies and solutions are currently proposed for the treatments of these diseases, whereas being limited to curbing the progress or managing symptoms in most cases^2,3^.

Aside from medical treatment through chemical processes, artificial devices are developed to improve the quality of life of individuals. To bring neuroprosthesis into realization, the behavior of biological neurons as well as its connection and interaction with artificial neural networks must be considered. To this end, investigation of the interaction of neuronal cell assemblies is required to understand and reproduce a specific behavior driven by intrinsic spontaneous activity. Additionally, long-term replacement of damaged brain areas with artificial devices implies understanding of their neurophysiological behaviors.

In this context, new therapeutic approaches and technologies are needed both to promote cell survival and regeneration of local circuits^4^ and restore long distance communication between disconnected brain regions and circuits^5^. Thus, characterization and modeling of biological neural networks^6,7^ are crucial to develop the new generation of neuroprostheses that mimics biological dynamics and provides adaptive stimulation at biological timescale based on the principle of electroceutics^8,9^.

Thanks to the new neuromorphic platforms, performing biohybrid experiments involving the bi-directional communication between a living system and its artificial counterpart is becoming more and more relevant not only for the development of neuromorphic biomedical devices^8,9^, but also to elucidate the mechanisms of information processing in the nervous system^10^. Recently, major progress has been made in the field of neuroprostheses^6,7^, so as neuromorphic devices are now capable of receiving and processing input while locally or remotely delivering their output either through electrical, chemical or optogenetic stimulation^11^.

A bi-directional interaction performed according to closed-loop architecture is mandatory to perform biohybrid experiments^6^. Closed-loop technologies has seen significant advancements, particularly in the realm of adaptive, personalized therapies. A notable development in this area is the focus on leveraging various sensing systems to enable therapies that can deliver closed-loop (i.e. adaptive) biomimetic inputs to dysfunctional neural circuits over time. This approach has shown particular progress in the treatment of Parkinson’s disease where adaptive deep brain stimulation devices that adjust stimulation parameters based on neural signals, showed improved efficacy and reduced side effects compared to traditional fixed-parameter devices^12^ going as far as restoring functions such as walking^13^. Another notable development is found in Brain-Computer Interfaces (BCIs) that have shown major improvements in enabling real-time adjustments to enhance system performance and user interaction based on closed-loop architectures, and finding applications in prosthetic control and rehabilitation for stroke patients^14^.

In the neuromorphic engineering research, SNNs are designed using two distinct approaches: bio-inspired or biomimetic. The former is widely used for applications such as computation and artificial intelligence^15^ using accelerated time simulation of simple neuron models. The latter uses complex neuron models operating at biological timescale to simulate neural network dynamics and/or performing biohybrid experiments^16^. To perform bi-directional biohybrid experiments and develop bioelectrical therapeutic solutions for health care like electroceutics^8,9,17^, real-time bio-physics interface and SNN processing are mandatory to ensure interaction at biological timescale^18,19^. However, most of current solutions for biomimetic SNN simulations are software-based tools such as NEURON^20^, NEST^21^ or Brian2^22^ and show significantly high computation time, especially for complex neuron models with synaptic plasticity. Hence, they are not suited for real-time emulation at millisecond time scale^23^ differently from hardware-based SNNs^18^, characterized by complex neurons and synapses and which allow temporal accuracy of the stimulation. Nevertheless, real-time stimulation and processing of biological data using biomimetic Spiking Neural Network (SNN) are still quite rare^24^.

Hardware-based SNNs are analog or digital. Analog SNN systems^18,25,26^ show lower power consumption than digital SNNs^27^. Research on memristor devices as synapses has also been explored^28–30^. In contrast, digital SNNs are more flexible thus more suited for prototyping while showing overall quicker design time, hence constituting the best choice for preliminary experiments and design of the new generation of neuroprosthetics.

The prominent SNNs hardware platforms are TrueNorth^31^, BrainScaleS-2^32^, SpiNNaker^33^ and Loihi^34^. TrueNorth uses a digital architecture but is inspired by the principles of analog computation, emphasizing low power consumption and parallel processing capabilities. BrainScaleS-2 is primarily using Leaky Integrate-and-Fire (LIF) neurons for exploring learning algorithms and plasticity mechanisms in neural networks. SpiNNaker provides real-time processing and offers flexibility in simulating different network configurations and neuron models including LIF and Hodgkin-Huxley (HH) but with a limited number of conductance-based currents. While some of these systems present mobile versions like^35^ for BrainScaleS-2, most of them primarily offer access via cloud-based services rather than direct physical access hence limiting direct integration into embedded closed-loop system outside of the provided server-based infrastructure. Prior work of the team^36^ presented a hardware-only implementation of a biomimetic SNN, but facing limitations in terms of network configuration flexibility, system integration and computation accuracy.

In this manuscript, we present BiœmuS standing for BIOmimetic EMUlation Single compartment, an accessible low-cost platform for real-time emulation of biomimetic SNNs characterized by detailed biophysical models of neurons and synapses within a fully customizable network. We will also showcase the versatility and user-friendliness of BiœmuS, specifically tailored for biohybrid experiments and guaranteeing a seamless system integration between the biological and its artificial counterpart in the context of closed-loop applications for electroceutical treatments.

## Results

### Adaptable integrated real-time biomimetic SNN

**Fig. 1:**
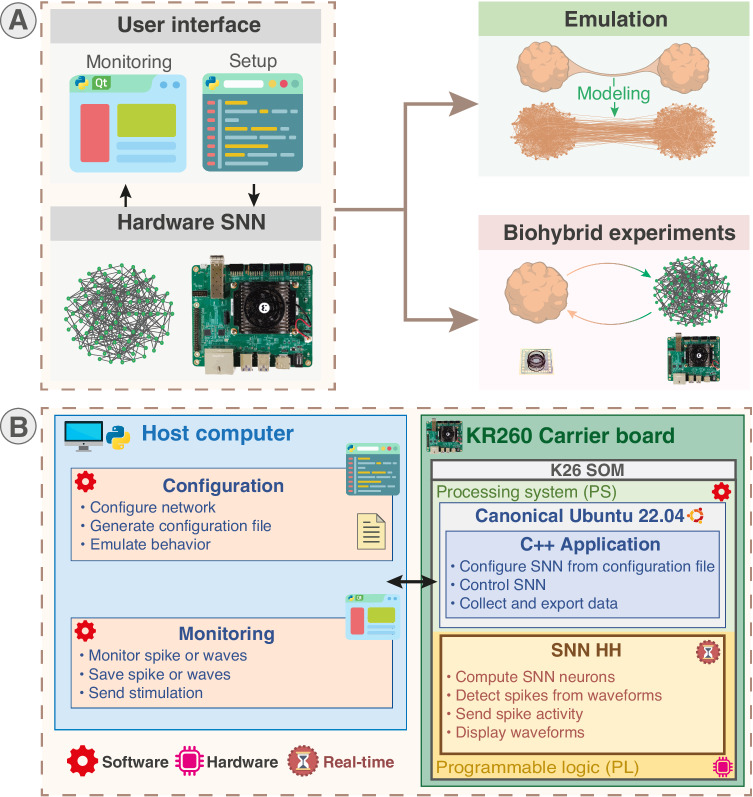
Overview of BiœmuS, a real-time hardware-based SNN for biophysically detailed emulation and biohybrid experiments. **A** BiœmuS corresponds to a platform integrating a hardware SNN allowing user to monitor and configure. the system through Python scripts and Qt-based GUI. The fully configurable network allows real-time emulation of custom network configurations that can be integrated in a biohybrid experimental setup acting as a versatile biomimetic artificial neural network easily interfaced with standard biological recording units. Network configurations displayed are exported from BiœmuS software. **B** BiœmuS runs on a carrier board integrating a System-on-Module featuring CPU in a Processing System part (PS) and FPGA in a Programmable Logic part (PL). The real-time hardware biomimetic SNN is implemented in PL part and controlled through a C++ application running in the PS part. The PS part runs a Canonical Ubuntu allowing standard interfacing and operation. Monitoring is performed by a Qt-based GUI and setup by Python scripts ran either on-board or on another computer.

The targeted low-cost platform is based on a System on a Chip (SoC) featuring both Programmable Logic (PL, i.e. FPGA) and processors in a Processing System (PS) part. It is capable of running up to 1024 HH neurons with fully configurable synapses, supporting a total of 2^20^ synapses. It features on-board monitoring and versatile external communication options such as Ethernet, Wi-Fi, expansion PMODs (standard peripheral module interface) and a Raspberry Pi header; allowing different compromises for monitoring and interconnection. The system allows real-time emulation of configurable networks as an accessible computing unit that can integrate biohybrid experiments with versatility (Fig. 1A).

The platform can be divided in two main parts referred to as software and hardware as identified in Fig. 1B. The hardware corresponds to the programmable logic part computing the Spiking Neural Network running in hard real-time at a period of 31.25 μs. The software part includes the configuration, monitoring and control running in soft real-time, implying fluctuating latency potentially larger than the deadline that is however not inducing a failure of the system, at a minimum period of 1 ms.

The neurons composing the SNN are modeled with high biological plausibility using the Hodgkin-Huxley (HH) paradigm^37^ in the Pospichil model^38^ implementing 6 conductance-based currents. An injected current mimicking synaptic noise following an Ornstein-Uhlenbeck process^39,40^ reproduces spontaneous activity by triggering action potentials on a random basis.

**Fig. 2:**
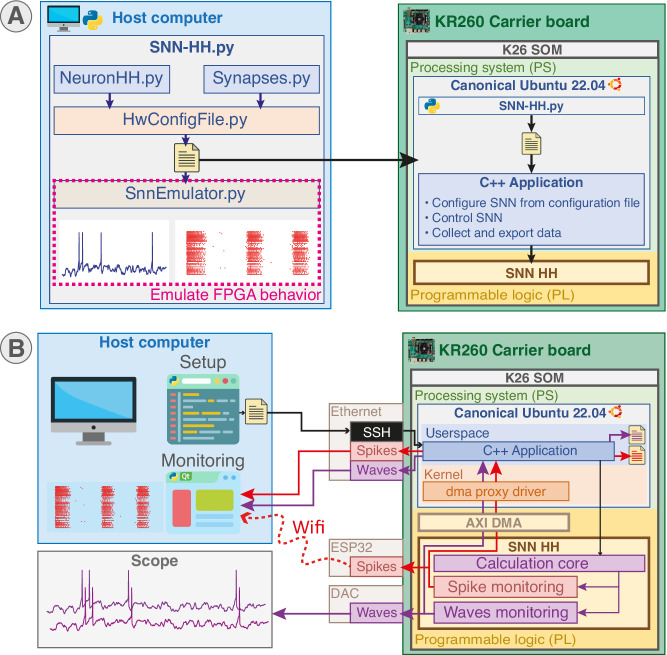
Architecture and system integration of the platform. **A** Overview of system setup from the configuration file generated by Python scripts ran either on-board or on another computer. The configuration file is then read by a C++ application running on Canonical Ubuntu operating system in the Processing System (PS) part to set up the SNN in Programmable Logic (PL) part. Configuration can be emulated beforehand to predict the behavior. Example membrane voltage and raster are exported from BiœmuS software. **B** Schematic of system communication. System control is achieved through the C++ application either remotely via SSH or directly on-board from the Ubuntu desktop. Spikes can be monitored concurrently using Ethernet, Wi-Fi and on-board file saving. Waveforms can be monitored concurrently using Ethernet, visualization on scope by probing the Digital-to-Analog Converter (DAC) and on-board file saving. Example membrane voltage is exported from BiœmuS software.

All parameters of the HH model as well as the synaptic noise parameters are tuned through the 25 parameters available from the Python scripts (Fig. 2A). The scripts implement 4 preset neuron types including Fast Spiking (FS), Regular Spiking (RS), Intrinsic Burst (IB) and Low Threshold Spiking (LTS) cortical neurons and allow the user to create new presets.

The equations of ion channel states are pre-computed and stored in memory so that they can be easily modified to any channel dynamic without impacting the performances of the system or limiting the mathematical functions used. The computation of ion currents is performed using 32-bit floating-point coding allowing emulation of currents with different dynamics potentially smaller in comparison to other currents like for Ca^2+^-based currents in IB or LTS neurons (Fig. S10B).

Neurons are connected using biomimetic synapses mimicking AMPA, NMDA, GABA_A_ and GABA_B_ synaptic receptors^41^ to allow fast and slow synaptic excitation or inhibition, computed using 18-bit fixed-point coding. The parameters of the synaptic models can be tuned similarly to the HH parameters through the Python scripts (Fig. 2A). Synaptic connection can be established between all neurons and independently weighted using the Python scripts allowing the user to create custom functions to setup the connections. The generated configuration file can be emulated using the Python scripts to assess behavior and verify membrane voltage, ion channel state equations, internal variables and raster plot (Fig. 2A).

To maximize compatibility and versatility, a Canonical Ubuntu is running on the processors of the board. Compatibility and versatility are important criteria, knowing that standards for communication protocols interfacing biological recording units vary along with manufacturers (e.g., Serial Peripheral Interface (SPI), Ethernet, USB). In addition, laboratories often have custom setups, designed to reach their specific needs or inherited from prior experimental settings. The selected carrier board features notably multiple USB3.0 and Ethernet ports as well as expansion PMODs (standard peripheral module interface) and Raspberry Pi headers, allowing implementation of a wide range of protocols.

The on-board monitoring allows storage of all spikes and up to 16 waveforms in a file, and also forwarding this data over Ethernet using ZeroMQ (as depicted in Fig. 2B), an open-source networking library widely utilized in embedded systems for its high performances and portability. Up to 8 membrane voltages of neurons can be selected at a time and output per Digital-to-Analog Converter (DAC) plugged on PMOD connectors. Data is moved from the PL to PS using Direct Memory Access (DMA) interfaced by Advanced eXtensible Interface (AXI) using a driver, thus providing high throughput and good scaling almost independently from the CPU load (Figs. S22, S23). The interval of collection and forwarding for spikes and waveforms can be set from the application settings.

A wireless setup communication for embedded applications is also provided via Wi-Fi using a PMOD ESP32 that plugs on PMOD connectors for spike monitoring. It communicates directly with the PL via SPI protocol driven by an ESP32 microcontroller that is able to receive and send data through Wi-Fi network (Fig. 2B). This solution offers a more flexible approach for interconnection of the system that suits well in vivo applications where cables are a concern, while maintaining a low latency and acceptable throughput. In addition, this constitutes a reusable element to build a reduced and minimal embedded version of the system targeting a smaller hardware-only target to create an energy-efficient solution for embedded applications.

The SNN is setup from the configuration file generated by Python scripts (Fig. 2A) that is either generated directly on-board using the Python interpreter installed on the Ubuntu operating system or on another computer. The application controlling the system can be launched directly on the board using the Ubuntu desktop or remotely over SSH (Fig. 2B).

The parameters of the application are generated in JSON format in a configuration file, allowing users to easily modify parameters without requiring code recompilation while also preserving parameters of the run. The parameters include the addresses for ZeroMQ forwarding, the local saving or other parameters such as the neurons to monitor (Table S3).

The firmware can be easily updated and loaded by running bash scripts, allowing convenient management of alternative versions developed for a custom dedicated hardware. An external stimulation trigger for each neuron with an independent duration is available via ZeroMQ to easily integrate the system in closed-loop setups.

### Example network configurations

This section demonstrates how the Spiking Neural Network of BiœmuS can be configured to be exploited for real-time emulation based on biological models using the provided Python scripts. Two examples of network configurations are demonstrated using in vitro and in vivo experimental models.

To offer a tangible example of network configuration for neurological disorders studies, in vitro cortical organoids have been selected as a demonstration case. Cortical organoids are three-dimensional tissue cultures derived from stem cells widely used to investigate the human brain properties. Thanks to their remarkable capability to replicate specific brain areas^42–44^, they allow for detailed studies of neural development and organization. Moreover, cortical organoids demonstrate functional maturation comparable to that observed in human, a feature not typically observed in traditional primary rat cultures^45^. This unique ability to recapitulate human brain-like properties makes cortical organoids an invaluable tool for advancing our understanding of neurodevelopmental processes and neurological disorders.

**Fig. 3:**
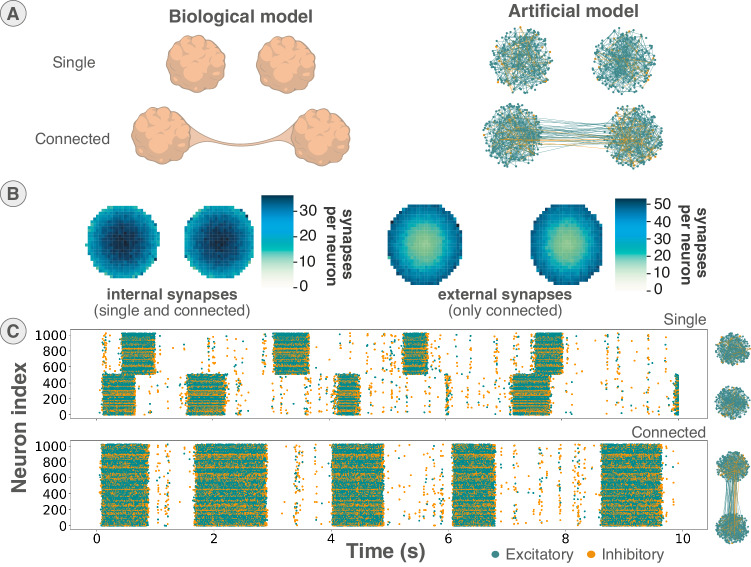
Example network configuration based on cortical organoids. **A** Two structures of cortical organoids modeled using FS and RS neurons connected with excitatory and inhibitory synaptic connection (AMPAR and GABA_A_R) based on biological culture observations and their spiking activity. Synaptic connections are promoted according to rules depending on the structure to reproduce, spatial placement of neurons and the ratio of inhibition/excitation connection observed. The network configuration was exported from Biœmus software. **B** Heatmap of the number of connection per neuron for the two structures of organoid interconnection showing internal and external synaptic connections. Internal connections correspond to the synaptic connections inside the two organoids of both structures in a closer neighbor connection manner. External connections correspond to synaptic connections between organoids for the connected structure, favoring synaptic connection on the exterior ring of the organoid. Heatmap are based on an average of 40 random generations of synapses for a given XY mapping. **C** Raster plot of 10-second emulated activity of a single and connected structure using BiœmuS, highlighting network burst synchronicity for the connected structure. The spiking activity emulated corresponds to a maximum probability for connection inside and outside the organoids of respectively 10% and 2% with 512 neurons per organoid and a 20% inhibition/excitatory neuron ratio.

The presented model is based on two types of structures according to a recent study^44^, promoting different synaptic connections between organoids as depicted in Fig. 3A. This model constitutes a good example to illustrate how the network can be configured according to custom synaptic connection rules defined by the user, to align with a specific model.

The structure referred to as “single” serves to physically isolate organoids, preventing any direct connections between them. This configuration functions as a reference model, allowing observation of the independent activity of each organoid. On the other hand, the “connected” organoids, or connectoid, positions organoids several millimeters apart while facilitating constrained interconnections, typically forming axon bundles that predominantly link neurons on the organoid surface^46,47^. This setup promotes stronger and more intricate biological activity^44^.

An additional Python class has been created for that specific model to assign normally distributed XY coordinates to neurons and generate synaptic connections based on specific rules for each structure. The synaptic connection probability inside organoids is set as a linear function of the relative distance between neurons, where the connection probability for the connected organoids is a linear function of their relative distance to the center of the organoid. The connectivity map obtained, shown in Fig. 3B, presents the connectivity map of internal and external synapses confirming the synaptic connection rules. The matrix of connection and list of neurons generated is then simply translated to hardware SNN configuration by the existing software (Fig. 2A), showcasing a case of custom user script to generate the network configuration.

The two structures were emulated using 1024 neurons distributed equally between the two organoids with a similar inhibitory/excitatory ratio to biology^44^. Inhibition is modeled using FS neurons connecting by GABA_A_R and excitation by RS neurons connecting by AMPAR.

While the number of neurons used is significantly lower, the emulation is able to reproduce from network bursts to the burst synchronization between organoids in the connected structure^44^ as shown in Fig. 3C.

An additional feature of the network configuration was explored by introducing specific synaptic receptor inhibition to mimic drug treatments, such as full antagonist to AMPAR (CNQX) and full antagonist to GABA_A_R (Bicuculine) (Fig. S14). The results obtained suggest that certain behaviors, such as epileptic seizures, could be replicated, but would require partial receptor inhibition rather than complete inhibition, or periodic inhibition over short durations.

**Fig. 4:**
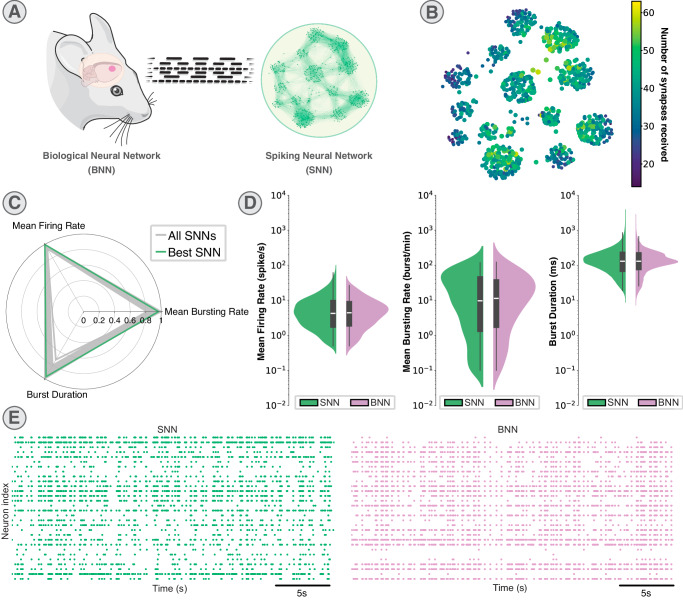
Example network configuration based on rostral forelimb area of rat brain. The data represented corresponds to a dataset comprising the 6 best Spiking Neural Network (SNN) among the 80 tested configurations and recordings of 6 Biological Neural Networks (BNN). **A** Representation of the BNN modeling according to a brain-like clustered network topology for the SNN. The network configuration was exported from BiœmuS software. The rodent head was cropped from the work of Servier (https://creativecommons.org/licenses/by/3.0/). **B** Synaptic connectivity map showing the number of the synapses received per neuron for the best configuration. **C** Radar plot comparing the fitting of the 80 SNN configurations to BNNs in terms of Mean Firing Rate (MFR), Mean Bursting Rate (MBR) and Burst Duration (BD). Highlighted in green are the 6 best SNNs (i.e. those configurations maximizing the similarity to the BNN group). **D** Distribution of the Mean Firing Rate (MFR), Mean Bursting Rate (MBR) and Burst Duration (BD) for the best SNN (977 neurons) and the 6 BNNs (282 neurons). Data are summarized in violin plots, where the outlines illustrate kernel probability density, i.e. the width of the shaded area represents the proportion of the data located there. Inside, the box plots (grey) where the horizontal lines denote the 25th and the 75th percentile values, the central white line the median value and the whiskers denote the 5th and the 95th percentile values. *p* = 0.9626 for MFR, *p* = 0.7944 for MBR, *p* = 0.7279 for BD non-parametric Mann–Whitney Rank Sum test (two-sided hypothesis test). **E** 30-s raster plots of the best SNN compared to the spiking activity of a representative BNN.

The second demonstration illustrates various network configurations aimed at recapitulating the electrophysiological features of an in vivo cortical biological network. The modeled network focuses on the Rostral Forelimb Area (RFA) region of the rat brain with the intention to exploit the artificial RFA to promote stimulation of the living brain (Fig. 4A). This bio-artificial interaction aims to facilitate adaptive intracortical microstimulation, delivered to a spared region, the Somatosensory area (S1), via the neuromorphic biomimetic SNN, to explore innovative strategies of open-loop electroceutical for brain repair as in^17^.

The network configurations utilize up to 1024 neurons distributed as FS neurons connecting with GABA_A_R for inhibition and RS neurons connecting with AMPAR for excitation, similarly to the previous example. However, this demonstration case introduces a network topology in a brain-like fashion, composed by a set of clusters with a higher density of connections within and fewer connections among them^48^, as depicted in Fig. 4B. The synaptic connection rule followed a close neighbor pattern. The parameters were swept to replicate the in vivo recorded activity. This illustrates how the network can be configured to adapt to a different connectivity model using the provided configuration scripts.

A total of 80 distinct configurations were simulated, varying parameters related to synaptic noise, synaptic weight, and the ratio of inhibitory/excitatory neurons. These configurations were then compared with the biological recordings according to three main biomarkers, specifically Mean Firing Rate (MFR), Mean Bursting Rate (MBR) and Burst Duration (BD) as summarized in Figs. 4C, D. Data was collected from the RFA area (i.e. the BNN) of 6 different Long Evans rats. Then, aforementioned metrics were computed for both the BNNs and the 80 SNNs, to derive a grade reflecting differences between two types of networks. From the radar plot of Fig. 4C, a subset of 6 SNN configurations was chosen, i.e. those which maximized the similarity to the BNNs.

The biomarkers related to these configurations were juxtaposed with the biological data according to MFR, MBR and BD in Fig. 4D. No significant difference between the SNN and the BNN was observed for the three metrics indicating the high level of performances of our SNN in emulating the activity of the BNN. The absence of statistical significance was demonstrated by performing a Mann–Whitney *U*-test (Table S5). Additionally, raster plot visualization for comparison was provided in Fig. 4E, highlighting similarity in the spiking activity between the best SNN and one representative BNN.

### Prototypes of biohybrid experiments

This section presents examples of biohybrid experiments using the system to interact with commonly used recording interfaces. It aims to show how different network configurations of the system from single neuron to larger network can interact with biological counterpart, whether in vitro or in vivo, through various interfaces.

**Fig. 5:**
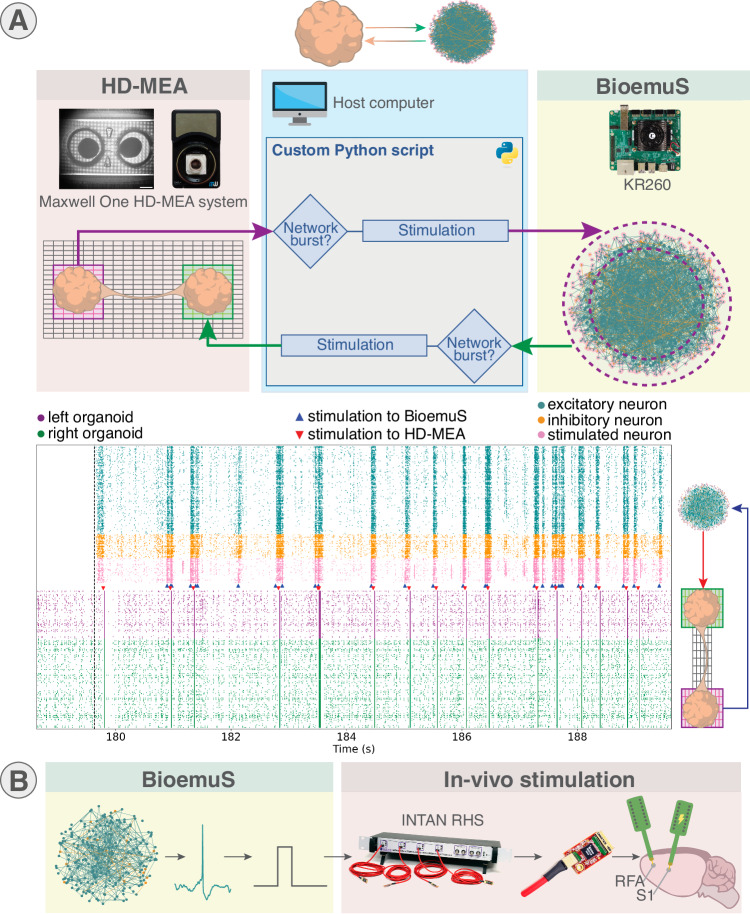
Prototypes of biohybrid setups integrating BiœmuS. **A** Closed-loop interaction between connected organoids plated on HD-MEA system and single organoid emulated on BiœmuS. The network burst detected in the left organoid of the connectoid triggers stimulation on exterior neurons of the emulated single organoid on BiœmuS. The network burst detected in the activity of BiœmuS triggers stimulation on the right organoid of the connectoid. Detection and stimulation commands are carried out in different threads. Stimulation of BiœmuS is performed using the external stimulation slot. BiœmuS stimulation triggers are shown by blue triangles and stimulation to HD-MEA by red triangles. BiœmuS is running for 180 s starting from 180 s and synchronized with HD-MEA activity from collected time stamps ± 100 ms. The raster plot is generated from python. The experiment was replicated 3 times with similar results using different detection threshold. **B** In vivo stimulation driven by BiœmuS spiking activity as a model of post stroke rehabilitation via biomimetic neural pattern stimulation. The spiking activity of the SNN triggers stimulation on an in vivo culture using the INTAN RHS2116 headstage. Electrode arrays were placed in the rostral forelimb area (RFA) and in the primary somatosensory area (S1) in the brain of adult Long-Evans rats. The experiment was replicated 5 times and the entire dataset has been analyzed with no discrimination among animals in^17^. The network configuration and action potentials were exported from Biœmus software.

A first demonstration case supporting the versatile integrability of the system with existing solutions for biological interfacing is a closed-loop stimulation between BiœmuS and the new generation of HD-MEA (High-Density MicroElectrode Array)^49^ (Fig. 5A). HD-MEA was selected for this demonstration case as a promising recording device garnering more attention and becoming more widespread in closed-loop setups^50,51^.

Connected organoids were plated on HD-MEA to interact with an artificial neural network and allow a clear delimitation between recording and stimulation sites. Electrodes were configured to allow activity recording on left and right organoids while allowing stimulation of the right organoid based on an activity scan and stimulation electrodes were manually selected among the ones exhibiting the most activity (Fig. S16). A single organoid was modeled using BiœmuS with a network of 1024 neurons emulated for 180 s. The closed-loop was controlled by a Python application running on a ‘gateway’ computer responsible for control, activity scan and stimulation, that will be referred to as the closed-loop software. Spiking activity of BiœmuS was forwarded to the gateway computer using ZeroMQ over Ethernet and stimulation was sent using ZeroMQ on the external stimulation port of BiœmuS. A stimulation was sent to the HD-MEA when a network burst occurs on BiœmuS.

The closed-loop software controlling both systems records activity of HD-MEA before and after the closed-loop for 180 s while collecting time stamps of stimulation triggers. This experiment showcases the potential of BiœmuS to operate as a tool to study the impact of adaptive stimulation on a culture following the principles of electroceutics, while highlighting its ability to adapt to a standard biophysical interface. The benefit of the user-defined model through customizable Python scripts to adapt to a specific application is also showcased here by assigning XY coordinates to neurons to take advantage of the spatial resolution provided by the HD-MEA.

The results obtained show that the closed-loop successfully triggers stimulation to both systems, however evaluating latencies and handling stimulation artifacts are limitations of the experiment setup. Precise synchronization of both systems would require additional development such as adding Precision Timing Protocol (PTP) to synchronize both system clocks or better characterization of the latency induced by the HD-MEA API as the latencies of BiœmuS already have been characterized (Figs. S18, S21, S22S23). As observed in Fig. 5A, stimulation artifacts are captured by the Maxlab Software, hence potentially inducing uncontrolled escalation as artifacts are considered as spiking activity.

A second type of interaction with the living thanks to the real-time behavior of BiœmuS is to drive an open-loop in vivo stimulation by the SNN^17^ as shown in Fig. 5B. This open-loop stimulation was applied to rat brains as a neuromorphic-based open-loop set-up for neuroprosthetic applications with the ultimate goal to be exploited in post-stroke rehabilitation studies^6,7^. The spikes from neurons emulated by BiœmuS become inputs to the INTAN RHS recording/stimulation unit to trigger stimulation in S1 (somatosensory area), to mimic the Activity Dependent Stimulation (ADS) paradigm in which spikes from RFA trigger stimulation in S1^52,53^.

The spontaneous behavior of the neurons is tuned to obtain slow or fast activities by tuning the parameters of the equation ruling the synaptic noise^17^. In this setup, the latency between spike detection in the SNN and stimulation is less than a millisecond.

### Performances

The low-cost platform targeted is the AMD Xilinx Kria KR260 Robotics Starter Kit carrier board embedding the K26 SOM by AMD Xilinx (Zynq Ultrascale+ MPSoC architecture). This entry level platform is capable of running 1024 neurons with 6 conductance-based currents for a total of 2^20^ conductance-based synapses running in hard real-time with a time step of 31.25 μs. The system can also run on AMD Xilinx Kria KR260 Vision Starter Kit carrier board with for only restriction the number of PMODs, preventing concurrent use of DAC waveforms and Wi-Fi spike monitoring. The system is also compatible with Petalinux, a lighter operating system for AMD Xilinx targets facilitating tuning of the Linux kernel to enable functionalities such as pre-emptive scheduling.

**Fig. 6:**
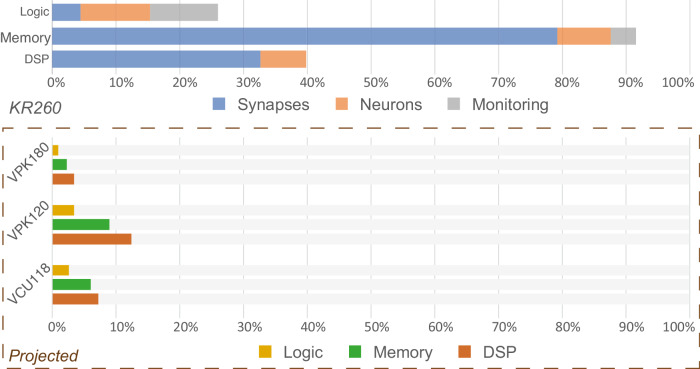
Hardware resource utilization. Utilization for main modules implemented on AMD Xilinx KR260 Robotic Starter Kit and projected on high-end evaluation boards from AMD Xilinx (Versal Premium Series VPK120 and VPK180 Evaluation Kits and Virtex UltraScale+ VCU118 Evaluation Kit). Logic; LUT and Flip-Flops. Memory; total memory implemented as BRAM and URAM. DSP; number of Digital Signal Processing (DSP) slices.

While most of the memory available is used, less than 50% of the computing capacity (Logic and Digital Signal Processing slices) of the board is used by the system (Fig. 6). As the design is implemented on an entry level target, the projection of the resources utilization on larger targets suggests the possibility to run several calculation cores in parallel (Fig. 6) as well as allowing faster emulation. The porting of the computation core is greatly facilitated by the use of High Level Synthesis design for computation modules allowing faster generation of optimized hardware for a given target.

The average latency observed to send spikes through ZeroMQ is typically under 500 μs (Fig. S23). The average latency observed for spike monitoring through Wi-Fi using ESP32 is between 0.792 ms and 2.098 ms (Fig. S21) depending on the data collection interval. The different paths of monitoring implemented allow finding compromises between throughput, latency and data integrity, providing a great flexibility in adapting to user requirements. Overall system power consumption is 6.50W with 3.42W associated with the calculation core (Fig. S20).

## Discussion

In this paper, we introduce BiœmuS, a low-cost, embedded, flexible and real-time biomimetic tool, which allows to perform biohybrid experiments for real-time emulation of living systems.

Our embedded system offers a real-time, cost-effective and user-friendly solution for closed-loop applications, addressing accessibility challenges prevalent in current high-performance alternatives. Unlike server-based infrastructures or complex systems like TrueNorth, SpiNNaker and BrainScaleS-2, which can be expensive and difficult to integrate into experimental setups, our solution prioritizes simplicity and accessibility. Notably, even software alternatives like Brian or NEURON, despite GPU acceleration, frequently struggle to achieve the low latencies necessary for closed-loop application.

Thanks to its straightforward setup facilitated by Python scripts, the system becomes easily accessible to neuroscientists, ensuring they can efficiently utilize its capabilities without being overwhelmed by technical difficulties. Additionally, thanks to the generic Ubuntu operating system handling communication, the system is capable of implementing a variety of complex protocols such as USB 3.0, USB 2.0 or Ethernet while maximizing software compatibility. Thus, our solution greatly facilitates integration, overcoming the usual drawback of time-consuming and technically intricate programming associated with low-level FPGA development. While non real-time operating systems as Ubuntu induce a discernible and fluctuating latency, using PL driven interrupt and AXI DMA allows obtaining relatively low latency about the tens of microseconds where the biological time is defined to one millisecond. A trade-off between latency and compatibility/versatility can be found by using solutions such as data sent directly by PL through expansion PMODs or ESP32, real-time operating system or running the application on the real-time cores of the chip. Nonetheless, direct monitoring on the PL that drastically reduces the latency remains possible using the various connectors of the board such as PMOD interface, GPIO or SFP.

On the current target, the main bottleneck lies in the memory usage essentially allocated for synapses weights and premultiplied rate tables for ion channel states. Since the current target is using a preceding architecture, more efficient architectures of memory can be found in recent larger targets such as High Bandwidth Memory (HBM) that integrates DRAM directly into the FPGA package, thus providing drastically higher depth and bandwidth. Latest AMD Xilinx chips also incorporate adaptive SoCs that provide significantly higher computation power (Fig. 6), notably with native floating-point DSP and AI engine while still embedding a Zynq for setup and control. Hence porting a similar architecture of SNN on these targets would significantly increase performances and create a promising alternative to standard GPU.

While the use of a complex neuron model limits the number of neurons that can be implemented, our system derives several benefits from this approach in various aspects. The use of the Hodgkin-Huxley (HH) model significantly facilitates interaction with neuroscientists, as it relates to biophysical parameters, unlike the simpler Leaky Integrate-and-Fire (LIF) or Izhikevich model. The system notably proved that the network is able to maximize fitting for specific parameters as highlighted by the rat brain network configurations or to replicate biological dynamics such as burst synchronisation between connected organoids. Even using generic parameters of FS and RS cortical neurons from^38^ and less neurons that the biological counterpart, network configurations maximizing fitting for specific biomarkers could be obtained by tuning the parameters of the network. Hence, the system can be suited for closed-loop experiments focusing on certain biomarkers, as in^17^, where the primary focus is the MFR.

The open-loop in vivo biohybrid experiment promoted the use of BiœmuS as a tool to investigate stroke rehabilitation with an electroceutic approach by providing stimulation following a biomimetic neural pattern. The preliminary results presented in^17^, that show efficiency to increase the firing activity of both RFA and S1 compared to the pre-stimulation condition thanks to the neural biomimetic stimulation pattern, suggest that the biomimetic SNN contribute to improve the rehabilitation. These results support the hypothesis suggesting that a neural biomimetic pattern more effectively entrains the network in response to stereotyped stimulation, making the population tend to be more responsive to incoming electrical stimuli, which is in line with recent human studies^54^. Hence, driving stimulation from a connected network instead of a single stochastic neuron with BiœmuS would allow further investigation of that hypothesis and more globally of stroke rehabilitation in an electroceutic approach by providing activity-dependent stimulation.

The current experimental setup for the closed-loop biohybrid experiment with the HD-MEA shows certain limitations. Nonetheless, thanks to the architecture of BiœmuS that runs a commercial Linux, solutions including more specific design directly controlling and transferring the data from the HD-MEA to the hardware part for real-time processing could be considered. Furthermore, this experimental setup benefits from a facilitated tuning of the stimulation patterns or protocols by changing the parameters of the different configuration files. The HD-MEA setup could be extended to implement closed-loop electroceutical interventions, emulating the dynamics of neurological disorders like Parkinson’s or ALS, ultimately aiming to restore normal biological function.

The system has proven its ease of integration demonstrated by the biohybrid experiments conducted on some of the most widespread biophysical interface with low-level communication protocol (pulse on digital output) as well as complex communication protocols (Wi-Fi and Ethernet). The ease of use and flexibility also have been particularly promoted by the examples of network configurations showing examples of different complex networks created from customizable Python scripts (Figs. 3, 4). The experiment in Fig. 5A also highlighted this feature by interfacing the BiœmuS to a biophysical interface using only Python scripts. While the accurate modeling of large network dynamics is constrained by the number of implemented neurons, the real-time processing capability of the SNN, coupled with interconnections at various latencies and throughputs, makes it a valuable tool for closed-loop setups.

The presented applications demonstrate the flexibility of BiœmuS in adapting to the study of various biological processes, including the potential for neuroprostheses replacement through closed-loop in vitro stimulation driven by BiœmuS (Fig. 5A) and post-stroke rehabilitation through in vivo stimulation (Fig. 5B).

## Methods

### SNN modeling

The neurons composing the network are modeled using the Hodgkin-Huxley paradigm^37^, the most biomimetic model considering the biophysical meaningfulness as an essential criterion^55^. The biophysically meaningful nature of the model being essential to relate parameters of the models to biology. The model of cortical neuron implemented is based on the Pospischil model^38^ that features 6 conductance-based currents allowing emulation of 4 classes of cortical neurons being “fast spiking” (FS), “regular spiking” (RS), “intrinsically bursting” (IB) and “low-threshold spike” (LTS). A current mimicking intrinsic noise observed in the brain following an Ornstein-Uhlenbeck process^39,40^ is injected to reproduce spontaneous activity by triggering action potentials on a biomimetic basis.

The different conductance-based synaptic receptors are modeled using the biologically meaningful Destexhe model^41^ allowing emulation of AMPAR, NMDAR, GABA_A_R and GABA_B_R.

The noise seeds are generated by the PS and sent through AXI LITE to the noise generator thus allowing true random seeds as well as fixed seeds for debugging.

The computation of ion channel states is based on ‘premultiplied’ HH rate function tables similarly to^56^, allowing computation of each HH variable from a single multiply and add from table values looked-up based on the membrane voltage (Eq. (1)). This method eliminates the FPGA-specific limitations for complex mathematical functions such as division and exponential. A trade-off between accuracy and resource utilization can be balanced with the size of memory implemented to store the rate function tables (Fig. S6).

1 $${x}_{n+1}={r}_{1}({V}_{n})\times {x}_{n}+{r}_{2}({V}_{n})$$

where, *x*_*n*+1_ and *x*_*n*_ are respectively the new and current value of the ion channel state, *V*_*n*_ is the membrane voltage at previous time step, *r*_1_ and *r*_2_ are the ion rate tables decoded from the membrane voltage.

Applied to common equations of ion channel states, the premultiplied tables can correspond to Eqs. (2), (3) and Eqs. (4), (5).

2 $${r}_{1}(V)=1-dt\times ({\alpha }_{x}(V)+{\beta }_{x}(V))$$

3 $${r}_{2}(V)=dt\times {\alpha }_{x}(V)$$

4 $${r}_{1}(V)=1-\frac{dt}{ta{u}_{x}(V)}$$

5 $${r}_{2}(V)=\frac{dt\times {x}_{\infty }(V)}{ta{u}_{x}(V)}$$

where, *r*_1_ and *r*_2_ are the pre-computed rate table for ion channel states decoded from the membrane voltage, *dt* the time step in ms, *tau*_*x*_, *x*_*∞*_, *α*_*x*_ and *β*_*x*_ the equation of the ion channel state depending on the formalism used.

The step and range of the tables are tunable from software but the generic rate table size is fixed in hardware to 2048 (1 BRAM) that provides a good compromise between accuracy and resource usage. The default range is set to −76 mV to 52 mV that covers the amplitude range for the preset neurons. Temporal discretization using a small time step compared to the dynamics is chosen to allow numerical solving using the Euler-Maruyama’s method.

### FPGA design

In PL part, the computation core is clocked at 400 MHz, the AXI communication to PS at 200 MHz and external components on PMOD connectors such as DAC and ESP32 at 50 MHz. The use of multiple clocks is justified by hardware limitations of components and blocks, multiple clocking allows all parts of the design to work close to their maximum to maximize performances. Crossing clock domain is handled by dual clock BRAM and FIFO for most critical signals, the remaining signals are either handled by double flip-flops or extended. The computation core is fully pipelined.

Computation of ion channels states and currents are encoded using 32-bit floating-point. It grants good stability and accuracy to the computation of ion channels that are critical parts of the neuron dynamics. Since ion currents can have different dynamics potentially smaller in comparison to other currents, floating-point coding is more suited for most computation and especially for multiplications (Fig. S10). Calculation of the current sum and Euler Murayama’s method are encoded using 32-bit fixed-point. Large fixed-point coding for sum operations allows to save resources and computation latency compared to floating-point, while guaranteeing consistent accuracy. The synaptic noise and synapses that have less critical accuracy or perform well with fixed-point coding are computed with 25 and 18 bits fixed-point encoding to fit the ranges of DSP slices. The injected stimulation current only involved in the final sum is coded using 32-bit fixed-point. Synaptic weight is coded on 14 bits and can be multiplied by a factor specified in software to mimic a larger network behavior.

The numerical solver used is the Euler-Maruyama with a small time step compared to the system dynamics to guaranty stability (31.25 μs)^36^. To maximize performances and limit resources usage, DSP of the boards were inferred using macros for most operations. The model is validated using Python implementation emulating both premultiplied rates and fixed-point coding.

### System monitoring and control

The PS part is running the Canonical Ubuntu 22.04 for ZynqMP architecture. The main application controlling the SNN is coded and compiled in C++11. Setup from the PS to the PL is implemented by AXI LITE controlled through /dev/mem in the C++ application.

Communication between the PL to PS is implemented using AXI DMA controlled by the the C++ application using the dma_proxy driver provided by AMD Xilinx. The application implements a thread for each AXI DMA channel and cyclic buffers for AXI DMA transfers.

The Ethernet communication implements ZeroMQ Push-Pull messaging pattern with a different port for each data (spikes, waveforms, and external stimulation) that can be set from the JSON configuration file.

The interval of data collection can be set from the JSON configuration file from 1 ms to 255 ms for spike collection via DMA, from 3.125 ms to 15 ms for the waveforms collections. The Wi-Fi connection is using UDP protocol and the data collection interval can be set from 2 ms to 20 ms. The spikes can be saved and stored using different formats. Sending formats are either binary encoded (one bit per neuron) or as a cumulative sum for each neuron. Saving formats are either binary encoded or comma separated.

The data collection interval for the spikes and waveforms through the DMA can impact the load of the application, especially with non binary streams that create dependence with the activity of the network. A small interval will generate more frequent write in file or frame sending thus increasing the CPU load. The limit corresponds to a data collection interval smaller than the writing or sending time of the frame therefore blocking the software in a thread.

The data collection interval for Wi-Fi forwarding is limited by the hardware and latency of the Wi-Fi protocol so as high intervals generate too large buffers and too small interval may generate packet losses.

DMA-based monitoring can run local saving and Ethernet forwarding concurrently in most cases with large data collections interval but may dysfunction on small intervals due to processor performances. Spikes and waveforms monitoring through DMA can run concurrently in separate threads but may also dysfunction on small data collection intervals due to processor performances.

Wi-Fi, DAC and DMA-based monitoring can run concurrently without impact on performances. Bash scripts are used to compile the software, update the firmware and launch the application.

An external stimulation controlled via Ethernet over ZeroMQ allows to send a stimulation of a given time to a given neuron by passing the stimulation duration for each neuron to the PL using the AXI DMA (Fig. S4).

### Network configuration

#### Connected cortical organoids

The “single” physically separates the organoids to prevent connection. The “connected organoids”, or connectoid, places organoids millimeters apart while constraining the interconnection to a channel of 150 μm width^46,47^. The emulation model implements cortical neurons using FS and RS types connected by AMPAR and GABA_A_R.

The synaptic connection rules for the synaptic connections inside organoids are ruled by Eq. (6) that favors connection to neurons close to each other normalised by the diameter of organoid. The connections between organoids are ruled by Eq. (7), relying on the location of neurons in the organoid to promote connections in the exterior ring.

6 $${p}_{single}={p}_{max}\times \left(1-\frac{{d}_{{n}_{pre},{n}_{post}}}{{r}_{org}}\right)$$

7 $${p}_{connectoid}={p}_{max}\times \frac{1}{2}\times \left(\frac{{d}_{{n}_{pre},or{g}_{pre}}}{{r}_{or{g}_{pre}}}+\frac{{d}_{{n}_{post},or{g}_{post}}}{{r}_{or{g}_{post}}}\right)$$

where *p*_max_ is the maximum probability of connection, *d* is the distance, *r* the radius, *n*_*pre*_ and *n*_*post*_ the pre-synaptic and post-synaptic neurons, *org*_*pre*_ and *org*_*post*_ the center of the pre-synaptic and post-synaptic organoids.

The recordings were performed using the MED64 system (Alpha MED Scientific) and electrical signals were recorded at 37 °C at 20,000 Hz sampling rate. The recording noise was eliminated by band-pass filter between 0.1 and 10,000 Hz during the measurement, then filtered offline with a band-pass filter (300–3000 Hz) for spike analysis. Spikes were considered using a threshold of ±5 *σ*, where *σ* represents the standard deviation of the baseline noise during quiescent periods. The characterization of the firing activity was conducted using Python. The analysis focused on Inter-Spike Interval (ISI, ms), Mean Firing Rate (MFR, spikes/s), Inter-burst interval (IBI, ms) and burst length (ms). The burst detection was performed according to^57^ using the string method (i.e. a max inter-spike interval of 100 ms and 5 ms as minimum number of intra-burst spikes).

#### Rat Rostral forelimb area

A total of six adult, male, Long-Evans rats (weight: 300–400 g, age: 2–4 months; Charles River Laboratories, Calco, LC, Italy) were used in this study. The experiments were conducted at the Animal Facility of the Italian Institute of Technology (IIT) in Genova, Italy, with prior authorization granted by the Italian Ministry of Health and Animal Care (Italy: authorization n. 509/2020-PR). For detailed surgical procedures, please refer to^58^.

Recording of wide-band signals from the rostral forelimb area (RFA) and primary somatosensory area (S1) were carried out using 16-channel microelectrode arrays (MEAs) (A4 × 4–5 mm-100-125-703-A16, NeuroNexus) connected to Intan RHS headstages. These MEAs were part of a bidirectional electrophysiology interface system equipped with 16 stimulation/amplifier channels linked to the probes.

All the preprocessing analyses of the in-vivo recordings, up to their spike sorting, were performed in MATLAB (The MathWorks, Natick, MA, USA). The ePhys data underwent preprocessing via a custom MATLAB pipeline, including a 4th order elliptic bandpass filter (300–3000 Hz) to eliminate low-frequency components. A power-based spike detection algorithm known as Stationary Wavelet-based Teager Energy Operator (SWTTEO)^59^ was employed to identify spikes from the filtered data. The SWTTEO involved two levels of Stationary Wavelet Transform (SWT) paired with the use of the Teager Energy Operator (TEO). The TEO outputs were smoothed, summed, and their combination was thresholded. Subsequently, multiunits were sorted using a mixture of skew-t distributions on PCA-extracted features, following the approach outlined in^60^.

The characterization of the firing activity of both the biological and artificial neural networks was performed using Python. The analysis focused on several biomarkers including Mean Firing Rate (MFR, spikes/s), Mean Bursting Rate (MBR, bursts/min) and Burst Duration (BD). The burst detection was performed according to^57^ using the string method (i.e. a max inter-spike interval of 100 ms and 5 ms as minimum number of intra-burst spikes).

The comparison with biological networks was graded as the absolute values of differences normalized considering the maximum and minimum values of each biomarker among all configurations, following Eq. (8):

8 $$Grade=1-\frac{| dif{f}_{i,m}| -\min (| dif{f}_{m}| )}{\max (| dif{f}_{i,m}| -\min (| dif{f}_{m}| ))}$$

where *diff*_*i*,*m*_ is the i-th difference between the m-th biomarker of the i-th BiœmuS network configuration and the target value of the same biomarker of the biological network. To evaluate statistically significant differences, among the two conditions (SNN group vs BNN group) we adopted the non-parametric Mann–Whitney *U*-test Figure. *P*-values were set at 0.005.

### Biohybrid experiments

#### Closed-loop biomimetically driven stimulation on HD-MEA

The bi-directional communication between BiœmuS and the HD-MEA system is ensured by Python scripts running on a gateway computer that will be referred to as the closed-loop application. The HD-MEA was configured to record from channels both from left and right organoid based on an activity scan and to select stimulation electrodes on the right organoid (Fig. S16). The HD-MEA is the MaxOne chip of MaxWell Biosystems AG^49^.

The spiking activity of the HD-MEA and BiœmuS were collected at a period of 1 ms. The spikes received from BiœmuS on the host computer are analysed to detect the occurrence of a network burst. The network burst detection is computed using a sliding window of the total activity of the network over a period of 10 ms. Upon burst detection, a stimulation consisting of a 500 μs pulse of amplitude 500 mV sent to the HD-MEA using custom Python script derived from manufacturer’s templates. The stimulation was chosen according to the values used in the work^50^ that proved efficacy on a similar setup.

The spikes received from the HD-MEA triggered stimulation on BiœmuS upon detection of a network burst. The network burst of the HD-MEA activity was calculated from a sliding window on 250 ms of activity. The stimulation was sent through Ethernet over ZeroMQ to the external stimulation port of BiœmuS to trigger a stimulation of duration 3.120 ms and amplitude 0.03 mA/cm^2^ to neurons on the exterior ring of the organoid.

The network burst latency was estimated to be inferior to 10 μs in most cases (Fig. S18). The closed-loop application ran a thread for each task of receiving spikes from HD-MEA, receiving spikes from BiœmuS, sending stimulation to Maxwell and sending stimulation to BiœmuS.

The activity recording of the HD-MEA was controlled by the closed-loop application. The closed-loop application sent a first stimulation trigger to BiœmuS to start the SNN in the same time as enabling the closed-loop. The activity of the HD-MEA was analyzed using the script provided by the manufacturer. The spiking activity of BiœmuS was recorded on-board. An interface executed in another thread was displaying the summed activity of both networks along with thresholds for visual control.

The electrode configuration of the HD-MEA was exported from the software. The XY configuration of neurons, network configuration and stimulated neurons of BiœmuS were exported from the Python scripts. Detection of bursts and spikes triggering stimulation for both HD-MEA and BiœmuS were reconstructed from the time stamps collected. The synchronization of both activities was done from time stamp collections with a certainty of 100 ms based on the fluctuating latency to start the recording of the HD-MEA activity.

#### Organoid cultures

The use of human iPS cells was approved by the Institute of Industrial Science, The University of Tokyo and cells were handled in accordance with approved protocols. Human iPS cells were obtained from the Riken Cell Bank (409B2, HPS0076) and maintained on ESC-qualified Matrigel-coated 12-well plates in mTeSR plus medium (STEMCELL Technologies) and passaged every 5–7 days using ReLeSR reagent (STEMCELL Technologies). Cortical connectoids were generated using previously reported protocol^44^. Briefly, hiPSCs were dissociated using TrypLE Express and 10,000 cells per well were seeded into U-bottom ultra-low attachment 96 well plate (Prime surface, Sumitomo bakelite) in mTeSR plus supplemented with 10 μM of Y-23632. 24h later, media was replaced with neural induction media (NIM), consisting of DMEM-F12 with HEPES, 15% (v/v) knockout serum replacement, 1% (v/v) minimal essential media non-essential amino acids (MEM-NEAA), and 1% (v/v) Glutamax, supplemented with 100 nM LDN-193189, 10 μM SB431542, and 5% (v/v) heat-inactivated FBS. On day 2, NIM was replaced without the supplement of FBS and changed every other day until day 10.

From day 10 to 18, culture medium was replaced and changed every other day with neural differentiation media 1 (NDM1), consisting of 1:1 mixture of DMEM/F12 with HEPES and Neurobasal medium, 0.5% (v/v) N2 supplement, 1% (v/v) B27 supplement without vitamin A, 1% (v/v) Glutamax, 0.5% (v/v) MEM-NEAA, 0.25 mg/ml human insulin solution, and 1% (v/v) Penicillin/Streptomycin/Amphotericin (PSA) (Sigma, A5955). On day 18, culture medium was replaced with neural differentiation media 2(NDM2), consisting of Neurobasal medium, 0.5% (v/v) N2 supplement, 1% (v/v) B27 supplement with vitamin A, 1% (v/v) Glutamax, 0.5% (v/v) MEM-NEAA, 0.25 mg/ml human insulin solution, 200 mM ascorbic acid, and 1% (v/v) PSA, supplemented with 20 ng/ml brain derived neurotrophic factor (BDNF). On day 28, culture media was replaced with Neural Maintenance Media (NMM) consisting of Neurobasal Medium, supplemented with 2% (v/v) B27 supplement with vitamin A, 1% (v/v) Glutamax, 1% (v/v) PSA and 20 ng/ml BDNF.

Cortical organoids were subjected to connectoid formation after at least 28 days in culture. Here, a custom-made microfluidic device containing two holes which are connected through a narrow channel were bonded on a CMOS-based HD-MEA (MaxOne+, Maxwell Biosystems). Microchannel of the microfluidic device was coated with 2% Matrigel (Corning) in DMEM/F12 for 1 h at room temperature (RT). Next, coating solution is replaced with NMM and an organoid is placed into each of the holes. Cells were kept at 37 °C and 5% CO2 and half media change was performed every 3–4 days for the duration of cell culture.

### Supplementary information

Supplementary Information

## Data Availability

All data that support the findings of this paper are presented within the paper and the Supplementary Materials. Additional data related to this paper may be requested from the authors. Data on animal experiments are available under restricted access which can be obtained by contacting the authors.

## Code availability

The code related to BiœmuS is available at https://github.com/Ceramic-Blue-Tim/bioemus and includes analysis and acquisition scripts. Due to proprietary ownership, we do not provide open access to the code related to the HD-MEA closed-loop experiment, access request will be evaluated on a case-by-case basis.
