# Supplementary material for: BiœmuS: A new tool for neurological disorders studies through real-time emulation and hybridization using biomimetic Spiking Neural Network
Source: Nat Commun. 2024 Jun 20;15:5142. doi: 10.1038/s41467-024-48905-x (PMC11190274; doi:10.1038/s41467-024-48905-x)
Supplement: Supplementary file 1 — Supplementary Information [file 41467_2024_48905_MOESM1_ESM.pdf]

Supplementary materials:

BiœmuS: A new tool for neurological disorders studies through real-time emulation and hybridization using biomimetic Spiking Neural Network

Romain Beaubois[1,2,3], Jérémy Cheslet[1,2,3], Tomoya Duenki[2,3,4,5], Giuseppe De Venuto[6], Marta Carè[6,7,8], Farad Khoyratee[1], Michela Chiappalone[6,7,8], Pascal Branchereau[9], Yoshiho Ikeuchi[2,3,5] and Timothée Levi[1*]

[1*]IMS, UMR5218, CNRS, University of Bordeaux, France.
[2]Institute of Industrial Science, The University of Tokyo, Japan.
[3]LIMMS, CNRS-Institute of Industrial Science, UMI 2820, The University of Tokyo, Japan.
[4]Department of Chemistry and Biotechnology, Graduate School of Engineering, The University of Tokyo, Japan.
[5]Institute for AI and Beyond, The University of Tokyo, Japan.
[6]DIBRIS, University of Genova, Italy.
[7]IRCCS, Ospedale Policlinico San Martino, Italy.
[8]Istituto Italiano di Tecnologia, Italy.
[9]INCIA, UMR5287, CNRS, University of Bordeaux, France.

*Corresponding author(s). E-mail(s): timothee.levi@u-bordeaux.fr;

This PDF includes

- Figures S1 to S23
- Tables S1 to S5

1 Supplementary Figures

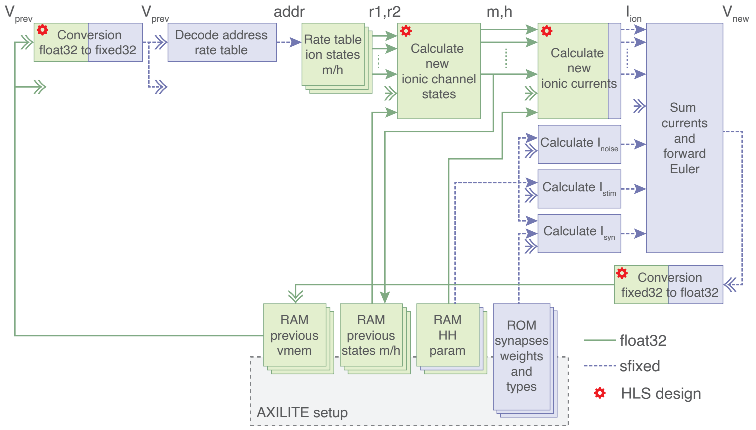

Fig. S1 Architecture of the calculation core in hardware.
The data encoding, floating-point or fixed-point, is shown for each module. Modules using floating-point operations were designed using Vitis HLS 2023.2. Initialization of the RAM and ROM is performed by the C++ application through AXI LITE.

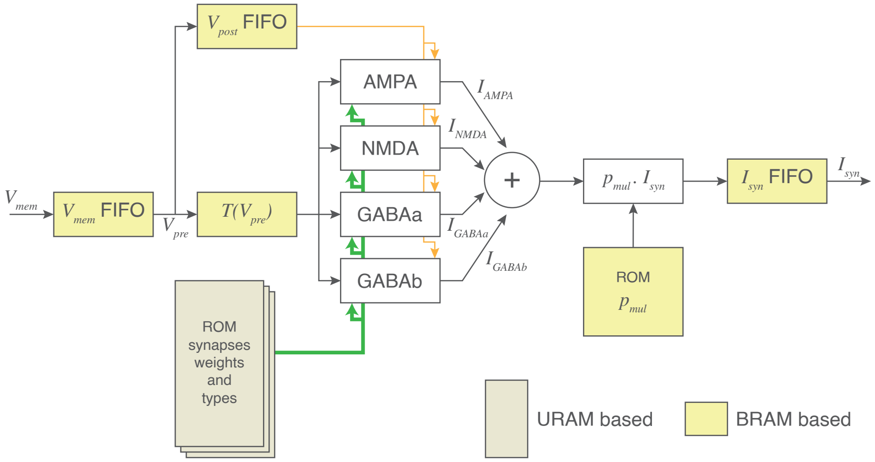

Fig. S2 Architecture of the synaptic current calculation module in hardware.
The type of memory implemented is shown for blocks integrating FIFOs, ROM or rate tables. The p_{mul} ROM corresponds to premultiplied coefficients including a scale factor to mimic larger network through stronger synaptic current.

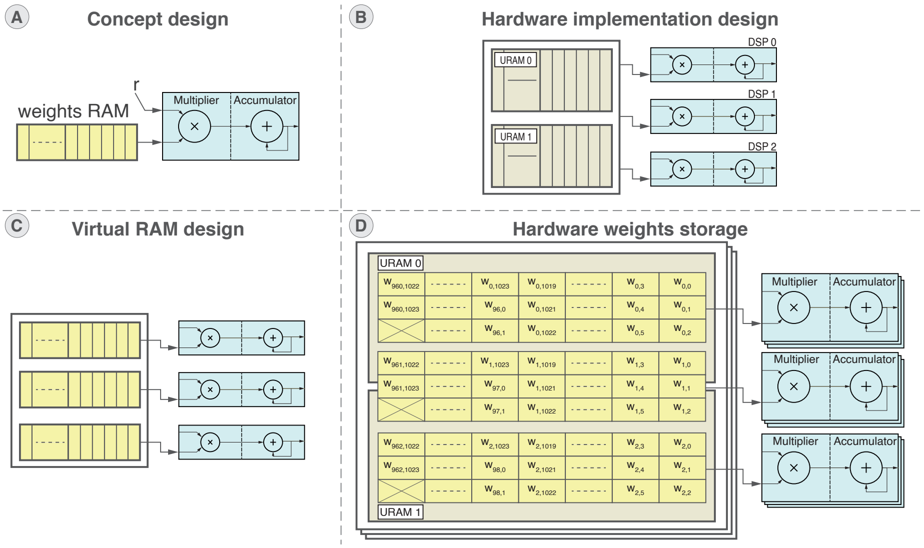

Fig. S3 Memory structure for synapses in hardware.
Concept and implemented block diagrams of calculations and memory organization for the synaptic current calculations. (**A**) Concept design of the synaptic current calculation where a DSP is configured in multiply-accumulate mode and connected to a RAM storing the weights. (**B**) Hardware implementation of (A) using URAM to store the synaptic weights (**C**) Virtual design of synaptic weights RAM that shows how RAM are interpreted for calculations. (**D**) Hardware implementation of (C) that presents the actual hardware storage of the weights distributed in multiple URAM.

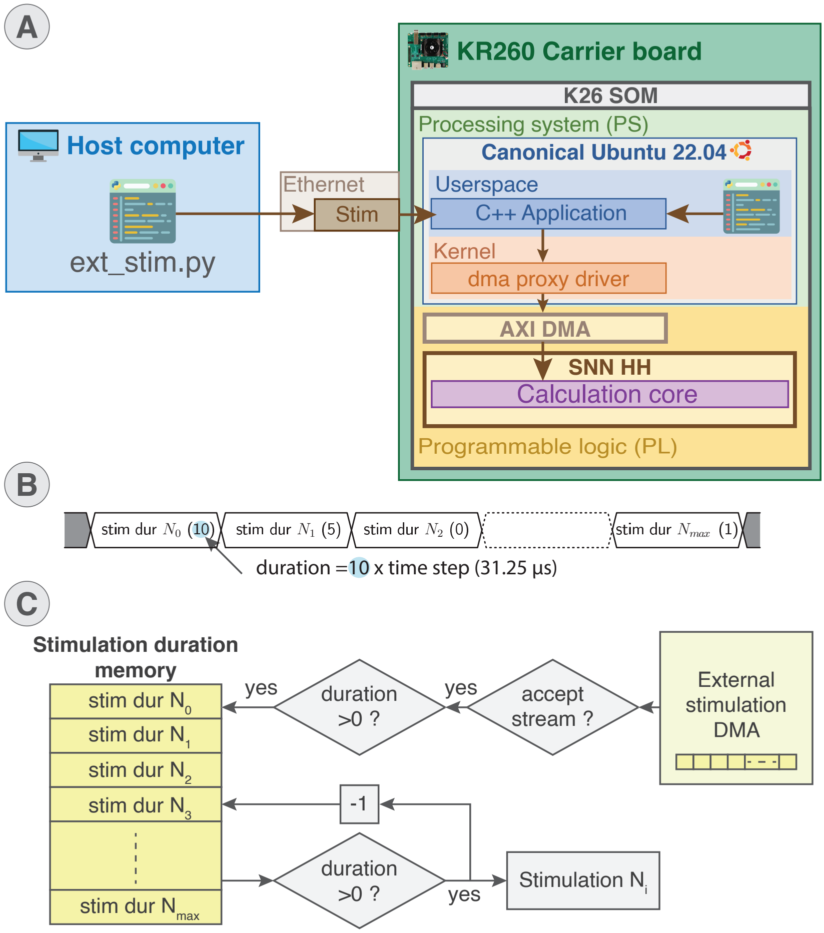

Fig. S4 External stimulation architecture.
Schematics and diagrams for external stimulation sent to hardware through AXI DMA. **(A)** Block diagram of the possible configurations for external stimulation. External stimulation is sent over ZeroMQ either through Ethernet from a remote host or locally on the target. The frame is forwarded using the AXI DMA controlled by a driver. **(B)** Frame structure of the external stimulation. The stimulation trigger corresponds to independent stimulation durations as number of time steps applied to given neurons. **(C)** Block diagram of the external stimulation module in hardware. The stimulation duration is set by the stream received from DMA

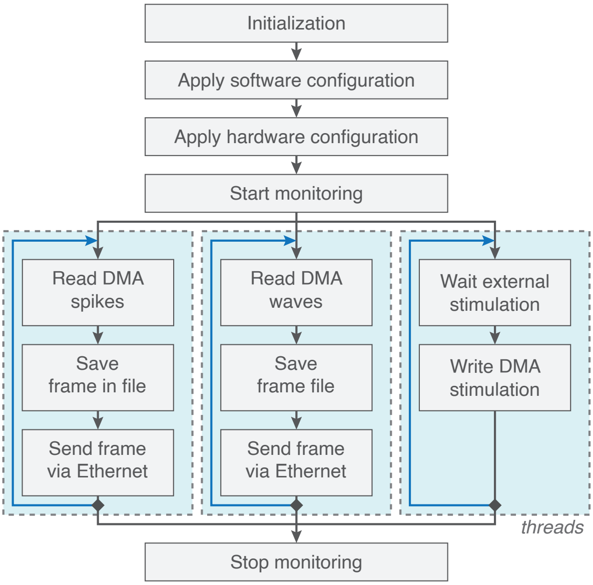

Fig. S5 High-Level workflow of the C++ application.
Monitoring of spikes and waves as well as external stimulation are operating in threads.

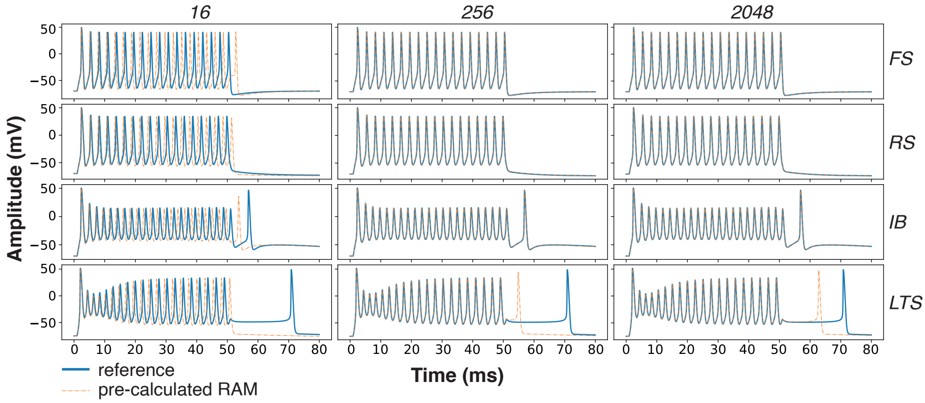

Fig. S6 Validation of premultiplied rates for ion channel states computation.
Comparison of the balance between the size of the pre-computed RAM (16, 256 and 2048) and its accuracy on the emulation of FS, RS, IB and LTS neurons in response to a 50 ms stimulation step of 0.03 $mA.cm^{-2}$. The simulation was performed in software with for sole difference the use of the premultiplied rates.

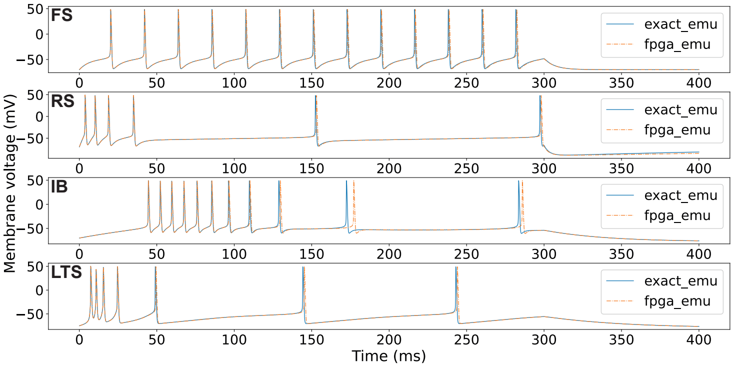

Fig. S7 Validating hardware implementation of neurons.
Comparison of membrane voltage response to a 300 ms current injection for Fast Spiking (FS), Regular Spiking (RS), Intrinsic Bursting (IB) and Low-Threshold Spiking (LTS) neurons for hardware implementation and reference emulation. exact_emu; reference emulation using exact equations for ion channels states, Euler-Murayama's method and 64-bit floating-point coding. fpga_emu; hardware emulation using premultiplied rates for ion channels, mixed data coding and Euler-Murayama's method. Waveforms were captured using the on-board saving with a collection interval of 14.375 ms.

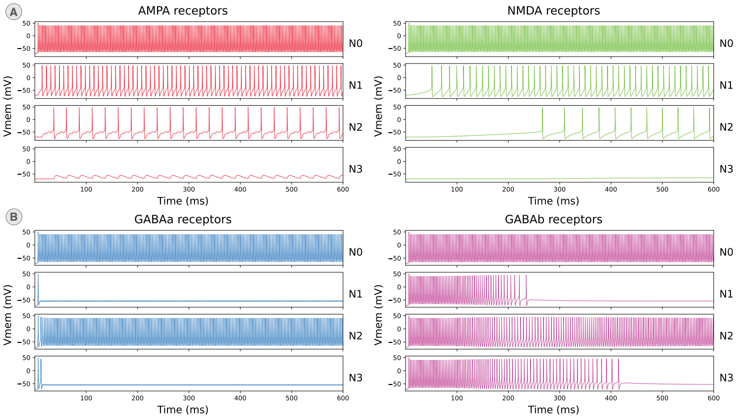

Fig. S8 Validating hardware implementation of synapses.
Behavior of a network of 4 identical neurons where only the type of synapses changes. The network connectivity is a chaser: neurons are interconnected with a single synapse, from N0 to N1, N1 to N2 and N2 to N3. A factor is applied to synaptic weights in order to see an excitation or an inhibition despite having only one incoming synapse on each neuron. **(A)** Only N0 is stimulated: AMPAR shows a faster response of excitation than NMDAR, synaptic weight of N2 to N3 is too small resulting in a lack of excitation of N3 thus preventing spiking. **(B)** All 4 neurons are stimulated: $GABA_AR$ shows a faster response of inhibition than $GABA_BR$.

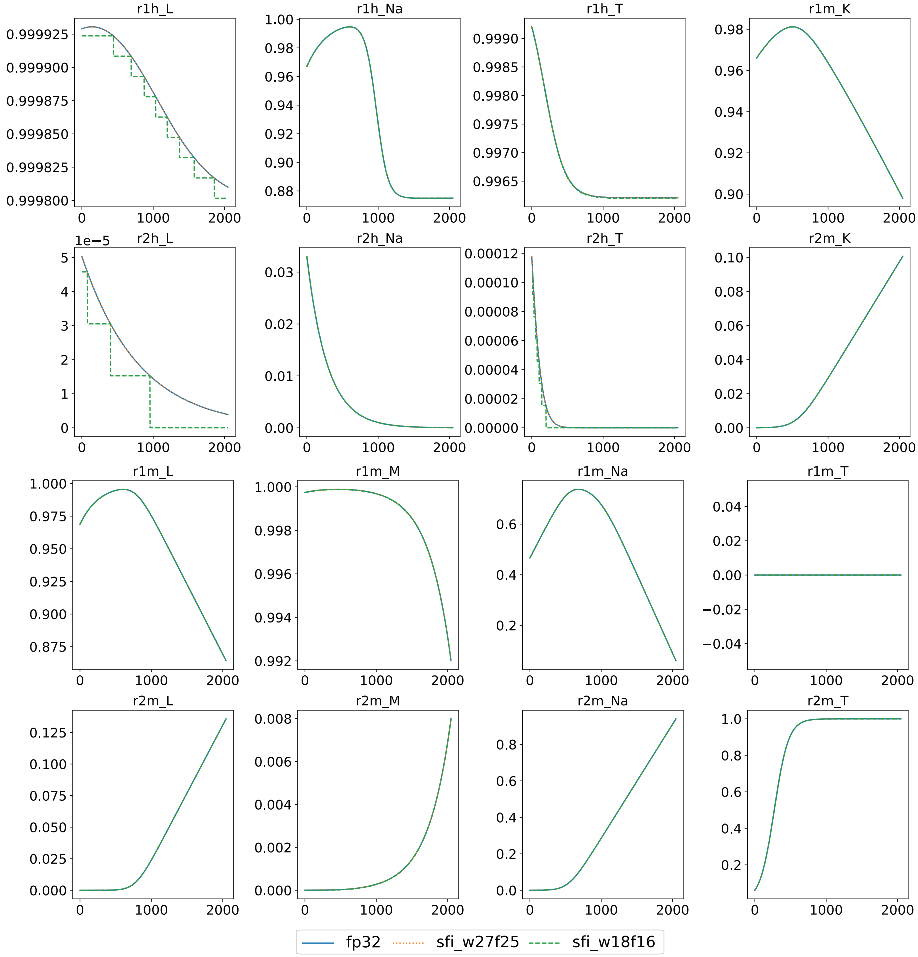

Fig. S9 Premultiplied rates for ion channel states using different data coding. Comparison of pre-computed rates stored in RAM for ion channel states using 18-bit fixed-point coding, 27-bit fixed point coding and floating-point coding. fp32; 32-bit floating-point. sfi_w27_f25; 27-bit signed fixed point with 25 bits of fractions. sfi_w18_f16; 18-bit signed fixed point with 16 bits of fractions. Labels corresponds to rate 1 or rate 2 for m or h states of ion channels. Rates that show small dynamics present significant accuracy loss when coded in 18-bit signed fixed point that is worsen by successive computations to obtain ion currents in the case of 18-bit fixed-point computation. Larger bits allocated enforces the use of more DSP to handle computations.

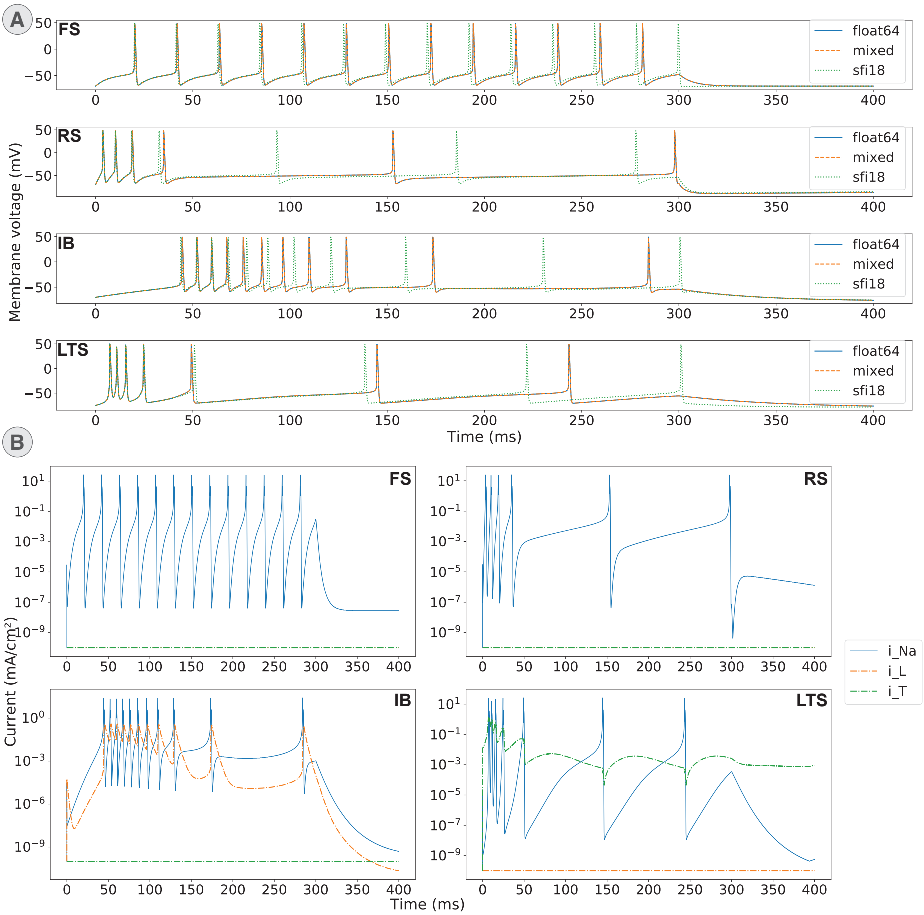

Fig. S10 Emulating neurons using different data coding.
Comparison between data codings for the computation of FS, RS, IB and LTS neurons emulated using Vivado HLS 2023.2. **(A)** Membrane voltage computation for four neurons types using different data coding for the complete computation in response to a 300 ms pulse. float64; reference implementation using 64-bit floating point. mixed; implementation used by BiœmuS using 32-bit floating point for most computation and 32-bit fixed point for the final sum. sfi18; implementation using 18-bit fixed-point with fractions adapted to variables ranges. Emulation were performed using Vivado HLS 2023.2 to accurately emulate the behavior of fixed-point in hardware as well as using the actual modules synthesized in BiœmuS. **(B)** Comparison of ion currents amplitudes in FS, RS, IB and LTS neurons using logarithmic scale. I_Na; sodium current. I_L; high-threshold calcium current. I_T; low-threshold calcium current.

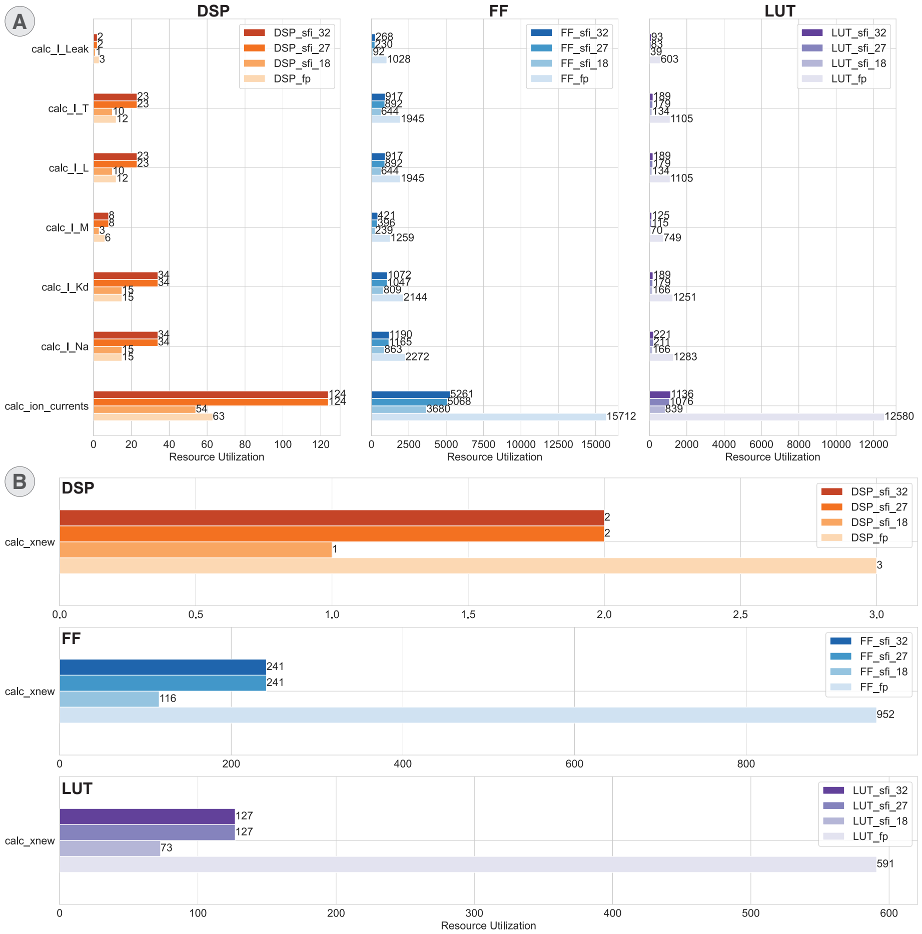

Fig. S11 Resource utilization for ion currents computation using different data codings.

Comparison of resource utilization for ion channel states and ion currents computations using fixed-point and floating-point coding synthesized with Vivado HLS 2023.2. DSP; Digital Signal Processing slices. LUT; look-up tables. FF; Flip-Flops. fp; 32-bit floating-point. sfi_32, sfi_27, sfi_18; 32-, 27-, 18-bit signed fixed-point. (**A**) Resource utilization of the module responsible for ion currents computations with sub-modules using different coding being 32-bit, 27-bit, 18-bit signed fixed-point and floating-point coding with outputs converted to 32-bit signed fixed-point. Larger signed fixed coding infers a larger number of DSP as it exceeds the maximum range of a DSP slice. Floating-point coding induces larger usage of logic for the computation and registers as the pipeline is longer. (**B**) Resource utilization of the module responsible for ion channel state computations from premultiplied rates using different coding being 32-bit, 27-bit, 18-bit signed fixed-point and floating-point coding.

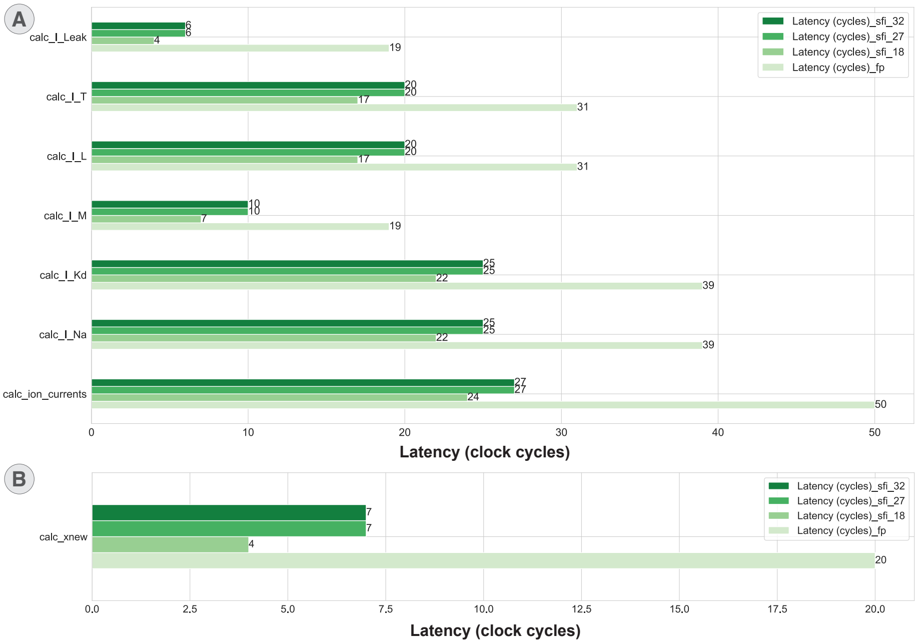

Fig. S12 Latency for ion currents computation using different data coding. Comparison of computation latencies for ion channel states and ion currents computations using fixed-point and floating-point coding synthesized with Vivado HLS 2023.2. Latencies are given in clock cycles and correspond to the latency of the pipeline. (**A**) Latency of the module responsible for ion currents computations with sub-modules using different coding being 32-bit, 27-bit, 18-bit signed fixed-point and floating-point coding. (**B**) Latency of the module responsible for ion channel state computations from premultiplied rates using different coding being 32-bit, 27-bit, 18-bit signed fixed and floating-point coding.

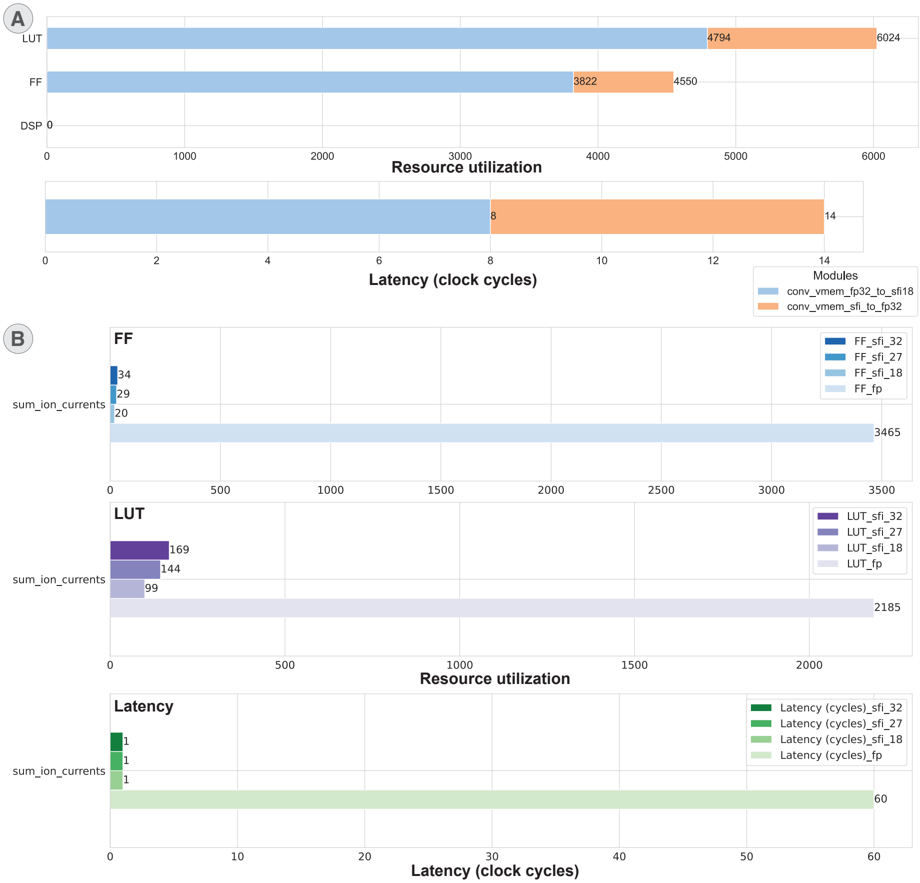

Fig. S13 Resource utilization and latency for floating-point and fixed-point coding conversion and sum of ion currents.
(A) Resource utilization and latency of floating-point and fixed-point conversions synthesized with Vivado HLS 2023.2. Floating-point to fixed-point conversion corresponds to the 6 ion currents conversions and fixed-point to floating-point conversion to the resulted value. Latency corresponds to the latency of the pipeline that is significantly low compared to the the clock cycles available per time step (12,500 clock cycles at 400MHz with a time step of 31.25 µs **(B)** Resource utilization and latency for ion currents sum using floating-point and fixed-point coding. Additions in fixed-point are faster, less resource intensive than floating-point and may have larger accuracy in terms of digits depending on fraction coding of the operands thanks to the absence of rounding error.)

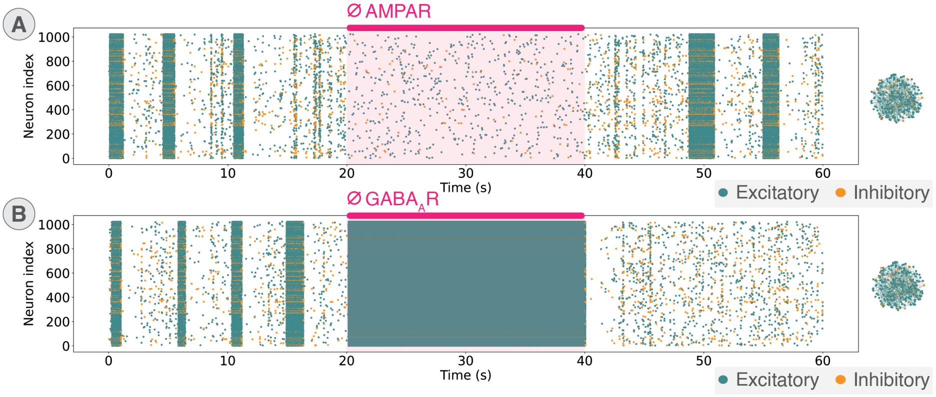

Fig. S14 Drug treatments emulations by synaptic receptors inhibition. Emulation of drug treatments in a single organoid through AMPAR and $GABA_A$R full antagonists from 20 seconds to 40 seconds. A trigger is sent to BiœmuS during emulation to disable a given receptor thus mimicking the drug treatment by full antagonist and a second trigger is sent to reactivate the receptor. The abrupt changes in spiking and bursting activity suggest that synaptic receptor inhibition should be done partially rather than completely. This setup was emulated once for each setup to confirm functionality of the synaptic receptor inhibition feature. **(A)** Emulation of treatment by full antagonist to AMPAR for 20 seconds prevents bursting and desynchronizes the activity. **(B)** Emulation of treatment by full antagonist full antagonist to $GABA_A$R for 20 seconds generates continuous spiking activity similar to an epilepsy seizure.

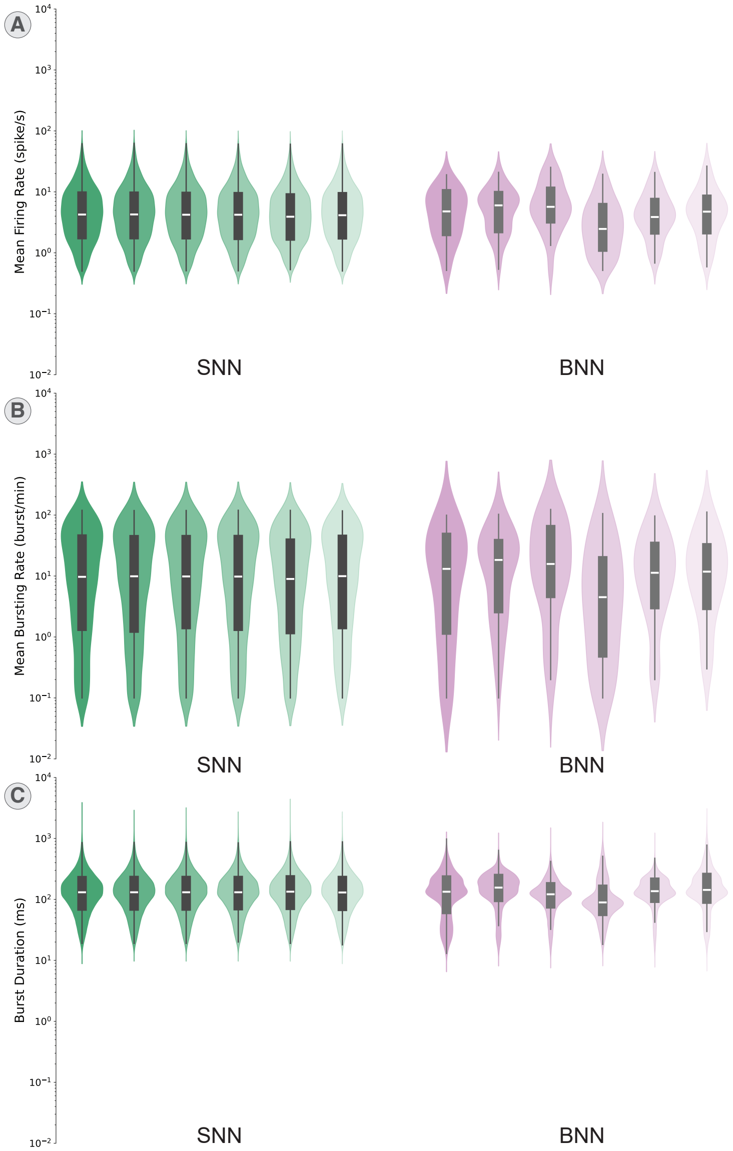

Fig. S15 Comparing spiking and bursting activity of SNN configurations with BNNs.
The SNNs correspond to the 6 best configurations obtained and the BNNs to the 6 recordings. **(A)** Violin plots of the Mean Firing Rate (MFR). **(B)** Violin plots of the Mean Bursting Rate (MBR). **(C)** Violin plots of the Burst Duration (BD).

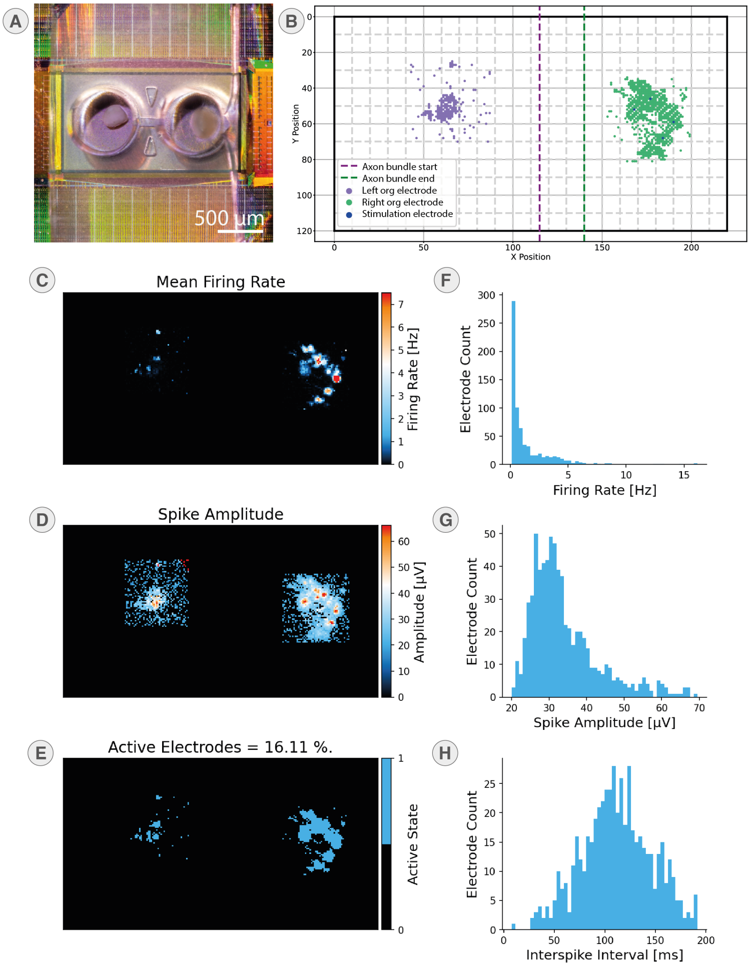

Fig. S16 Setup configuration for closed-loop stimulation on HD-MEA. Experiment and recordings were performed on organoids at week 40. The same organoids were used for all the experiments. **(A)** Connected organoids on MaxOne HD-MEA. **(B)** Electrode configuration of the HD-MEA chip for the experiments. Electrodes configuration is based on an activity scan carried out with MaxLab Live Software. Four electrodes of the right organoid exhibiting the most spiking activity are selected manually. Axon bundle start and end lines are selected manually. **(C)** Mean firing rate map of the connected organoids showing higher activity in the right organoid. **(D)** Spike amplitude map of the connected organoids. **(E)** Active electrodes map of the connected organoids. **(F)** Firing rates by electrode. **(G)** Spike amplitudes by electrode. **(H)** Interspike Interval by electrode.

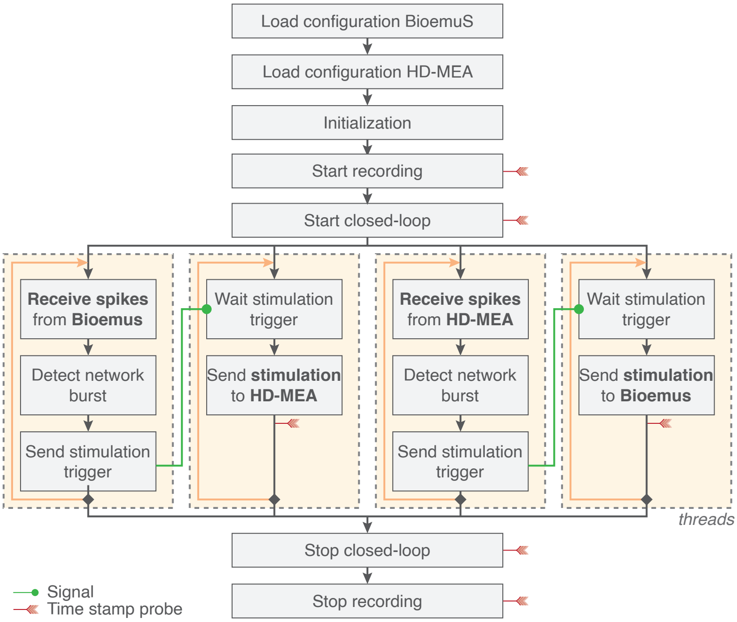

Fig. S17 High-Level workflow of the Python application controlling the closed-loop HD-MEA experiment.
The activity of BNN and ANN are scanned in concurrent threads that send a trigger to stimulation threads upon threshold crossing. Stimulation trigger is passed directly between threads using pyqtsignal. Time stamps are collected at each stimulation trigger and at the different stages of the experiment (recording before closed-loop, recording after closed-loop). The additional thread for the monitoring of the spiking activity of the network is not shown.

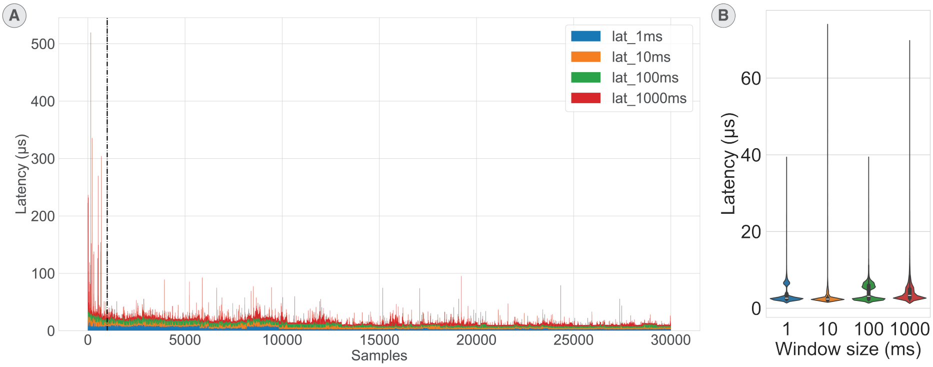

Fig. S18 Computation latency for network burst detection using Python.
Computation latency for network burst detection evaluated for different window sizes using Python. Test were performed using Python 3.11 on Intel Core i7-8565U. **(A)** Computation latencies for each sample using different window size. The black line delimits the starting point considered for analysis. **(B)** Violin plot of the latency observed from the start line shown in (A). n=29,000.

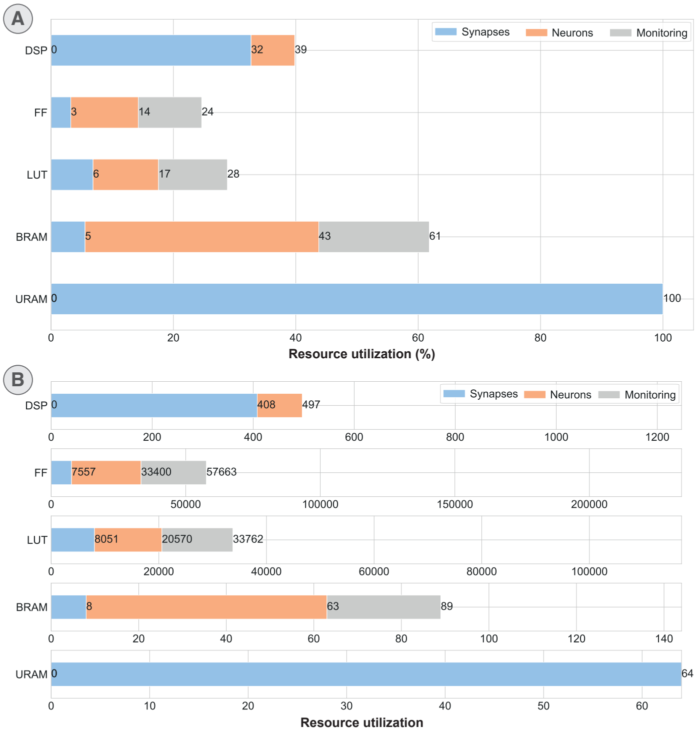

Fig. S19 Resource utilization of programmable logic (FPGA).
Distributed resource utilization for implementation on AMD Xilinx KR260 Robotic Starter Kit exported from Vivado 2023.2. **(A)** Percentage of resource utilization per category. **(B)** Resource utilization per category.

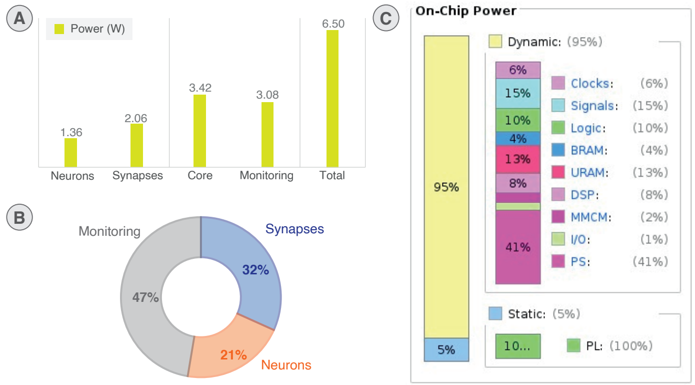

Fig. S20 Power consumption reports.
Power consumption for implementation on AMD Xilinx KR260 Robotic Starter Kit exported from Vivado 2023.2. **(A)**Power consumption by modules. *Core*; computation core that computes neurons and synapses. *Monitoring*; DMA along with the CPU cores. **(B)**Contribution in power consumption for main modules. While the computation core and monitoring show similar consumption, the synapses show larger power consumption than neurons. **(C)**Detailed power consumption report. PS; Processing system, mostly relating to CPU and memory.

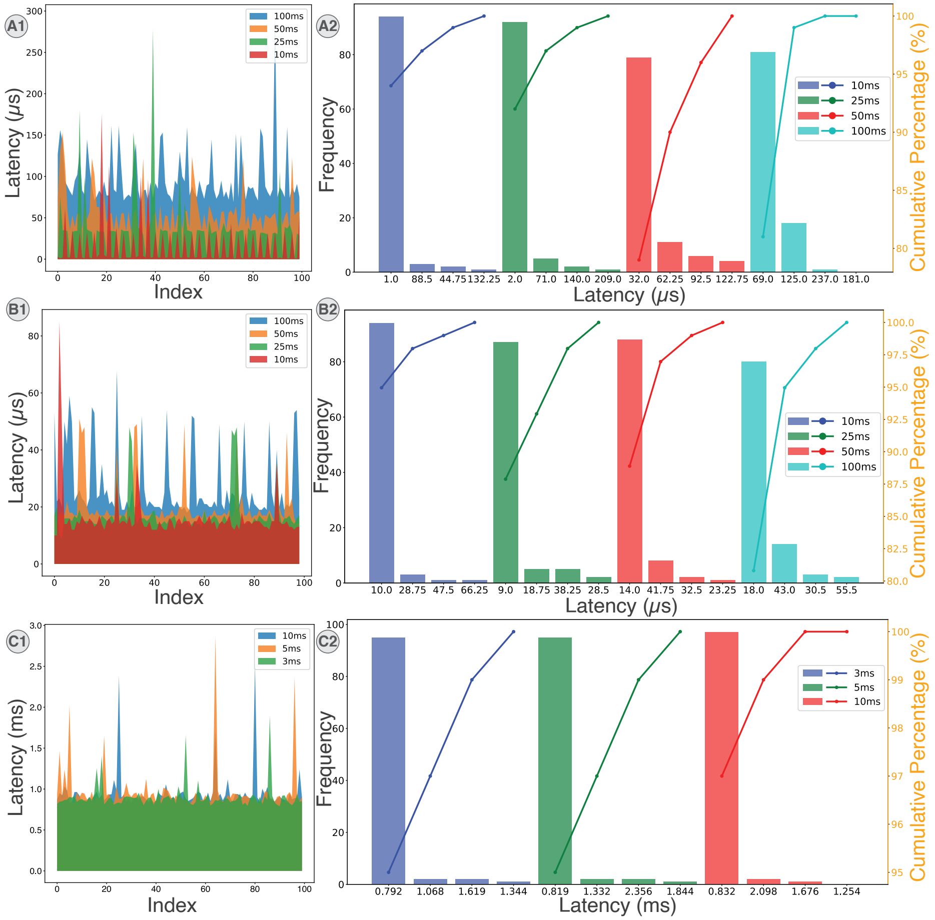

Fig. S21 Latency charts of spike monitoring.
Latency charts for spike monitoring for 100 data collections at different intervals. **(A1,A2)** On-board file saving latency for spikes coded in binary based on 100 samples at different data collection intervals represented sequentially and in Pareto charts. **(B1,B2)** Latency in application to send spikes with ZeroMQ over Ethernet for spikes coded in binary based on 100 samples at different data collection intervals represented sequentially and in Pareto charts. **(C1,C2)** Latency to send spikes over Wi-Fi between two ESP32 for spikes coded in binary based on 100 samples at different data collection intervals represented sequentially and in Pareto charts.

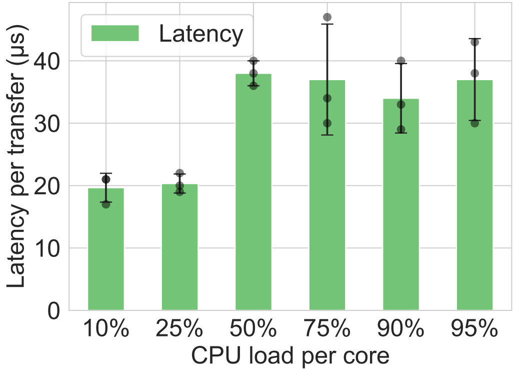

Fig. S22 Latency of dma_proxy driver.
Average latency per transfer of dma_proxy driver depending on CPU load with Ubuntu 22.04. This latency corresponds to the transfer of data from the hardware (programmable logic) to the software layer (userspace of Ubuntu). The tests corresponded to 4,096 transfers of 1,024 bytes on a hardware design implementing an AXI DMA in loopback clocked at 200 MHz. The average latency per transfer was obtained using the tests scripts provided by AMD Xilinx along with the driver code. Error bars show standard deviation. n=3 measures. CPU load was generated using CPULoadGenerator (https://github.com/GaetanoCarlucci/CPULoadGenerator)

.

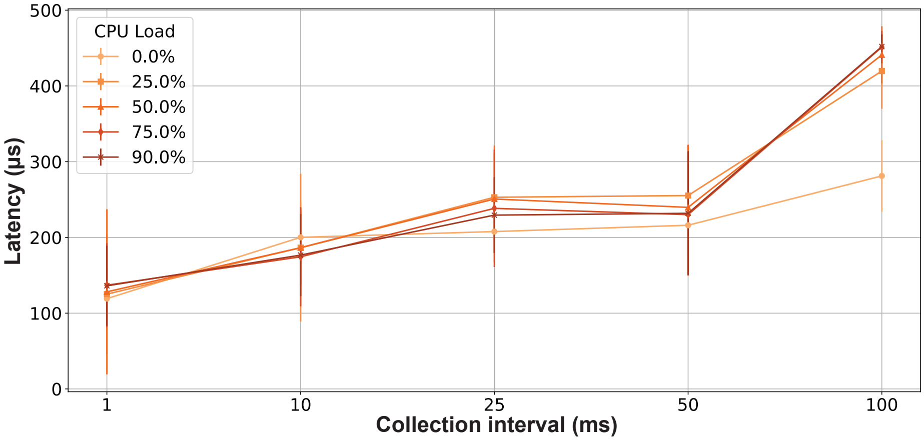

Fig. S23 Latency of ZeroMQ external stimulation and spike monitoring.
Average latency for local spike collection over ZeroMQ depending on CPU load for different collection intervals. CPU load was generated on all 4 cores using CPULoadGenerator (https://github.com/GaetanoCarlucci/CPULoadGenerator). Spikes were collected for a duration of 60 seconds using different collection intervals. The processors are Quad-core Arm® Cortex®-A53 MPCore™ with frequency governor set to performance (1.333 GHz).

2 Supplementary Tables

Table S1 Tunable parameters from the Python scripts and data coding in the system.

Parameter	Coding	Unit
		Neurons
g_{Na}	float32	S/cm^2
g_K	float32	S/cm^2
g_M	float32	S/cm^2
g_L	float32	S/cm^2
g_T	float32	S/cm^2
g_{Leak}	float32	S/cm^2
E_{Na}	float32	mV
E_K	float32	mV
E_{Ca}	float32	mV
E_{Leak}	float32	mV
v_{init}	float32	mV
μ_{noise}	sfixed18	1
θ_{noise}	sfixed18	1
σ_{noise}	sfixed18	1
I_{stim}	sfixed32	mA/cm^2
$area$	float32	cm^2
c_{mem}	float32	$\mu F/cm^2$
		Ionic channels[1]
m_{Na}	float32	1
m_K	float32	1
m_M	float32	1
m_L	float32	1
m_T	float32	1
h_{Na}	float32	1
h_L	float32	1
h_T	float32	1
		Synapses
w_{syn}	sfixed14	1
g_{AMPA}	sfixed18	nS
g_{NMDA}	sfixed18	nS
g_{GABA_a}	sfixed18	nS
g_{GABA_b}	sfixed18	nS
α_{AMPA}	ufixed17	$M^{-1}.s^{-1}$
α_{NMDA}	ufixed17	s^{-1}
α_{GABA_a}	ufixed17	$M^{-1}.s^{-1}$
β_{AMPA}	ufixed17	s^{-1}
β_{NMDA}	ufixed17	$M^{-1}.s^{-1}$
β_{GABA_a}	ufixed17	s^{-1}
$K1_{GABA_b}$	ufixed17	$M^{-1}.s^{-1}$
$K2_{GABA_b}$	ufixed17	s^{-1}
$K3_{GABA_b}$	ufixed17	s^{-1}
$K4_{GABA_b}$	ufixed17	s^{-1}
Kd_{GABA_b}	ufixed18	1
T_V[1]	ufixed17	M
B_V[1]	ufixed11	1

Max,min and step for float32 are not relevant since limited by model stability.

[1]Tunable through the equation stored in the premultiplied rate tables

Table S2 Parameters of the Hodgkin-Huxley model for the 4 preset neuron types tunable from the Python scripts.

Parameter	FS	RS	IB	LTS	Unit
g_{Na}	0.05	0.05	0.05	0.05	S/cm^2
g_K	0.01	0.005	0.005	0.005	S/cm^2
g_M	0.0	7×10^{-5}	3×10^{-5}	3×10^{-5}	S/cm^2
g_L	0.0	0.0	1×10^{-4}	0.0	S/cm^2
g_T	0.0	0.0	0.0	4×10^{-4}	S/cm^2
g_{Leak}	0.00015	0.0001	1×10^{-5}	1×10^{-5}	S/cm^2
E_{Na}	50.0	50.0	50.0	50.0	mV
E_K	-100.0	-100.0	-90.0	-100.0	mV
E_{Ca}	0.0	0.0	120.0	120.0	mV
E_{Leak}	-70.0	-70.0	-70.0	-75.0	mV
v_{init}	-70.0	-70.0	-70.0	-75.0	mV
μ_{noise}	0.048	0.042	0.042	0.042	1
θ_{noise}	8.0	8.0	8.0	8.0	1
σ_{noise}	0.11	0.09	0.09	0.09	1
I_{stim}	0.003	0.01	0.0006	0.0006	mA/cm^2
$area$	$(67 \times 10^{-4})^2$	$(96 \times 10^{-4})^2$	$(96 \times 10^{-4})^2$	$(96 \times 10^{-4})^2$	cm^2
c_{mem}	1.0	1.0	1.0	1.0	$\mu F/cm^2$

Table S3 Configuration parameters of the C++ application setup from the JSON configuration file.

Key	Description
fpath_hwconfig	Path to hardware configuration file
emulation_time_s	Emulation time from 1 to 2^{32} s by 1 s
sel_nrn_vmem_dac	List of 8 neuron waveforms to select on DAC
sel_nrn_vmem_dma	List of 16 neuron waveforms to select on DMA
save_local_spikes	Enable/disable spike local saving
save_local_vmem	Enable/disable waveform local saving
save_path	Path to local saving file
en_zmq_spikes	Enable/disable ZeroMQ spike sending
en_zmq_vmem	Enable/disable ZeroMQ waveforms sending
en_zmq_stim	Enable/disable ZeroMQ external stimulation
en_wifi_spikes	Enable/disable Wi-Fi spike sending
ip_zmq_spikes	IP address ZeroMQ spike sending
ip_zmq_vmem	IP address ZeroMQ waveform sending
ip_zmq_stim	IP address ZeroMQ external stimulation
bin_fmt_send_spikes	Select spike sending mode (binary or cumulative sum)
bin_fmt_save_spikes	Select spike saving mode (binary or csv)
nb_tstamp_per_spk_transfer	Time stamps to wait for spike collection (x1 ms)
nb_tstep_per_vmem_transfer	Time steps to wait for waveform collection (x31.25 µs)
en_stim	Enable/Disable simulation step
stim_delay_ms	Stimulation step delay in ms
stim_duration_ms	Stimulation step duration in ms

Table S4 Comparison of fitting performances for six different network configurations.

Network	MFR	MBR	BD	Final grade
SNN1	0.99921	0.96386	0.96870	0.93294
SNN2	0.99759	0.96536	0.96870	0.93289
SNN3	0.99564	0.96687	0.96870	0.93252
SNN4	1.00000	0.96837	0.96087	0.93048
SNN5	0.99525	0.96084	0.96870	0.92634
SNN6	0.99370	0.93825	0.99217	0.92504

Table S5 Mann-Whitney U-test of violin plots for the network configuration of the RFA.

Biomarker	p-value	Test result	Rank sum statistic	z-statistic test
Mean Firing rate	0.9626	0	615763	0.0469
Mean Bursting Rate	0.7944	0	490916	-0.2606
Burst Duration	0.7279	0	3.1226e10	0.3479

Non-parametric Mann-Whitney Rank Sum test (two-sided hypothesis test). P-values: p=0.9626 Mean Firing Rate, p=0.7944 Mean Bursting Rate, p=0.7279 Burst Duration. Test result: 1 indicates rejection of the null hypothesis; 0 indicates otherwise. Rank sum statistic: Value of the rank sum test statistic. z-statistic test: z-statistic to compute the approximate p-value of the test.
